# Supplementary material for: Gram-scale total synthesis of teixobactin promoting binding mode study and discovery of more potent antibiotics
Source: Nat Commun. 2019 Jul 22;10:3268. doi: 10.1038/s41467-019-11211-y (PMC6646333; doi:10.1038/s41467-019-11211-y)
Supplement: Supplementary file 1 — Supplementary Information [file 41467_2019_11211_MOESM1_ESM.pdf]

Gram-scale Total Synthesis of Teixobactin Promoting Binding Mode Study and Discovery of More Potent Antibiotics

Zong et al.

Supplementary Figures

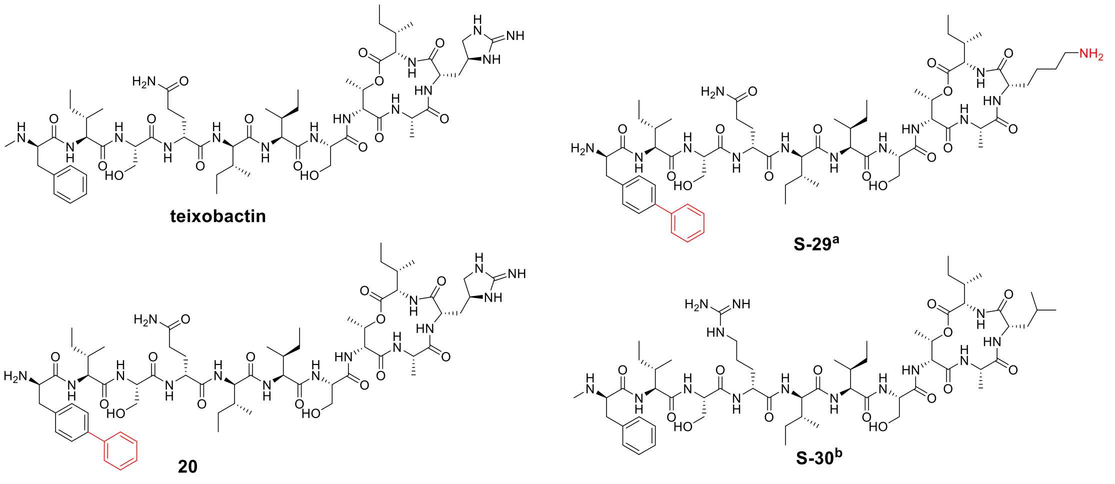

Supplementary Figure 1: structure of teixobactin and teixobactin analogs. a) **S-29** was prepared according to reference 1; b) compound **S-30** was prepared according to reference 2.

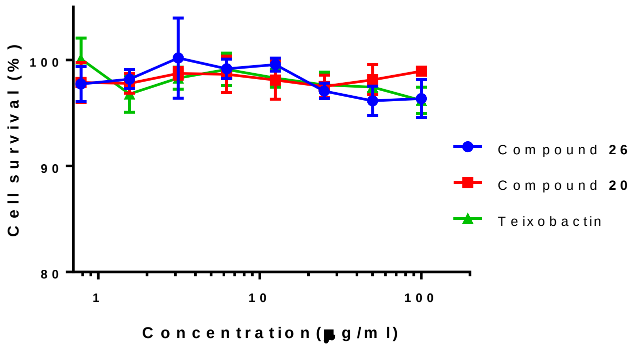

Supplementary Figure 2: The cytotoxicity of compounds **20** and **26.** The cytotoxicity of compounds **20** and **26** was evaluated in HepG2 cell line. Data represents 3 independent experiments ± s.d. (n = 3). Source data are provided as a Source Data file.

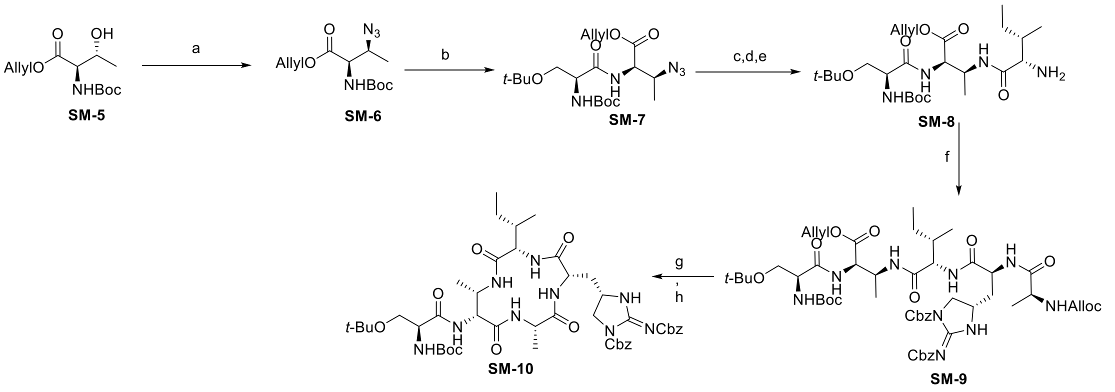

Supplementary Figure 3: Synthesis of lactam scaffold instead of lactone. **Conditions:** a) 1.2 equiv. trifluoromethanesulfonic anhydride, 1.3 equiv. 2,6-lutidine, -78 °C, then 2.5 equiv. tetrabutylammonium azide (n-Bu_4N_3), 23 °C, 1.5 h 85%; b) 30% TFA, r.t. 15 min, then 1.25 equiv. Boc-Ser(*t*Bu)-OH, 1.25 equiv. HATU, 3 equiv. DIEA, r.t. 3 h, 80% ; c) 4 equiv. PMe_3, THF:H_2O/9:1, rt, 12 h; d) 1.3 equiv. Fmoc-Ile-OH, 1.3 equiv. HATU, 1.5

equiv. DIEA, DCM:DMF/9:1, 3 h; e) 33% Et_2NH in MeCN, 15 min, 36%; f) 1.3 equiv. compound **14**, 1.6 equiv. DEPBT, 1.6 equiv. DIEA, THF/DMF, 30 °C, overnight, 53% for 2 steps; g) 0.2 equiv. $Pd(PPh_3)_4$, 2 equiv. 1,3-dimethylbarbituric acid, DCM, 30 °C, 1 h; h) 4 equiv. HATU, 4 equiv. HOAT, 8 equiv. DIEA, DCM/DMF, 30 °C, 24 h, 50%.

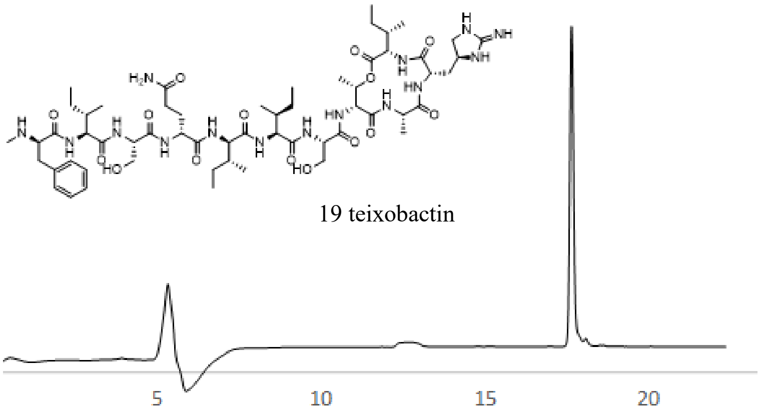

Supplementary Figure 4: HPLC spectrum of teixobactin. Gradient: 20-60% CH_3CN/H_2O with 0.1% TFA over 30 min at a flow rate of 3 mL/min. Wavelength (nm):214nm.

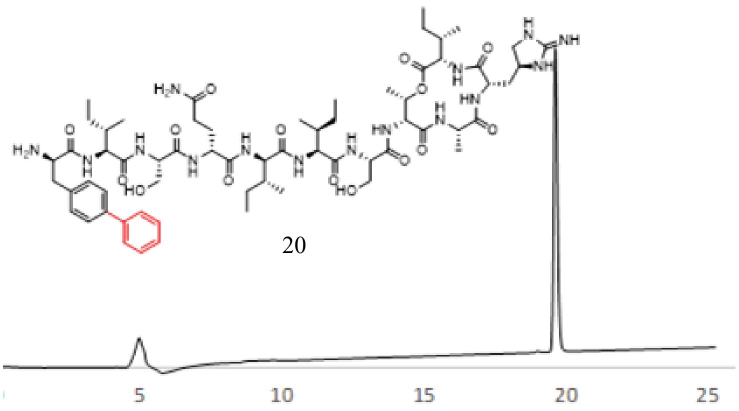

Supplementary Figure 5: HPLC spectrum of compound 20. Gradient: 20-60% CH_3CN/H_2O with 0.1% TFA over 30 min at a flow rate of 3 mL/min. Wavelength (nm):214nm.

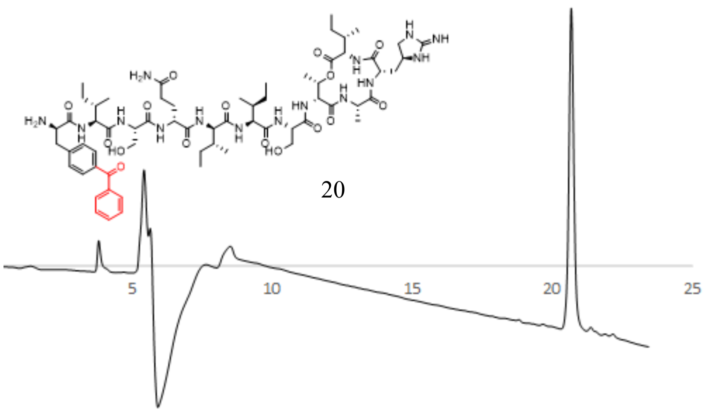

Supplementary Figure 6: HPLC spectrum of compound 21. Gradient: 20-60% CH_3CN/H_2O with 0.1% TFA over 30 min at a flow rate of 3 mL/min. Wavelength (nm):214nm.

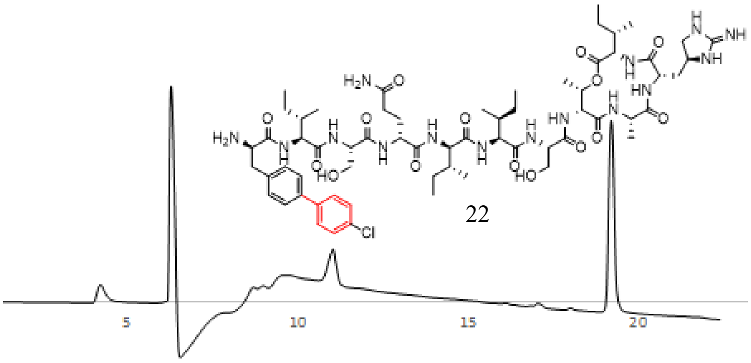

Supplementary Figure 7: HPLC spectrum of compound 22. Gradient: 1-91% CH_3CN/H_2O with 0.1% TFA over 25 min at a flow rate of 3 mL/min. Wavelength (nm):214nm.

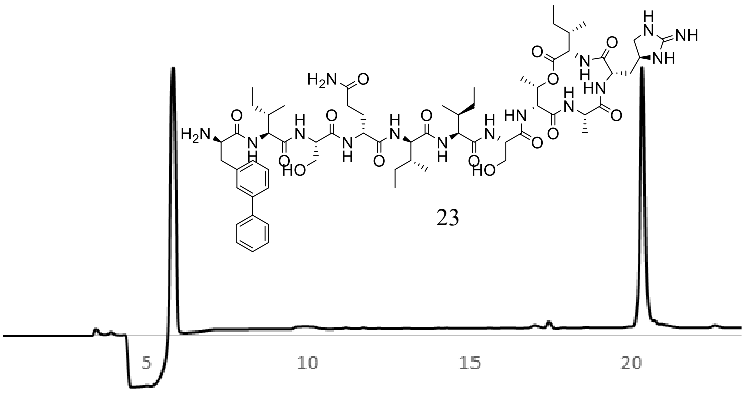

Supplementary Figure 8: HPLC spectrum of compound 23. Gradient: 20-60% CH_3CN/H_2O with 0.1% TFA over 30 min at a flow rate of 3.5 mL/min. Wavelength (nm):214nm.

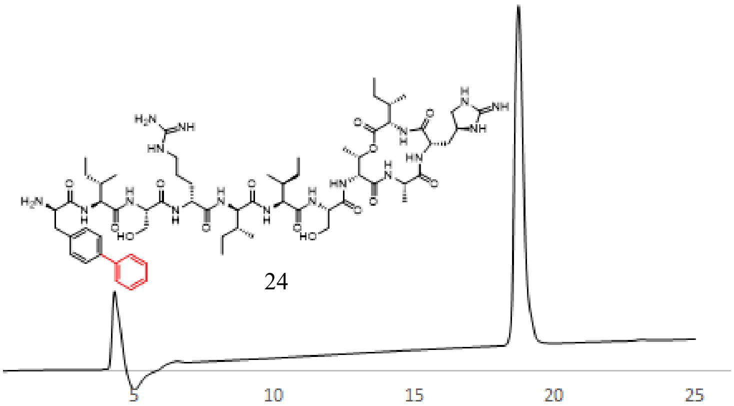

Supplementary Figure 9: HPLC spectrum of compound 24. Gradient: 20-60% CH_3CN/H_2O with 0.1% TFA over 30 min at a flow rate of 3.5 mL/min. Wavelength (nm):214nm.

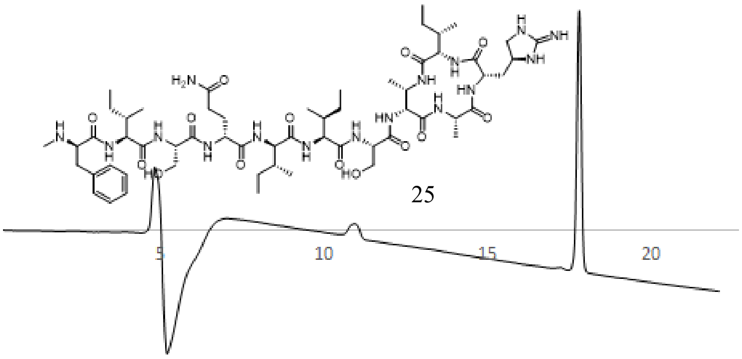

Supplementary Figure 10: HPLC spectrum of compound 25. Gradient: 20-60% CH_3CN/H_2O with 0.1% TFA over 25 min at a flow rate of 3.5 mL/min. Wavelength (nm):214nm.

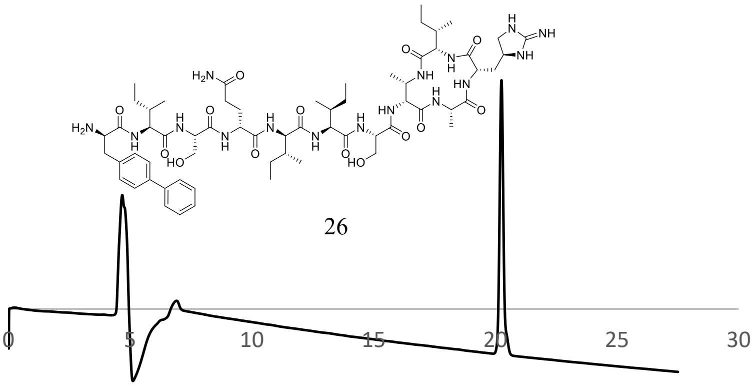

Supplementary Figure 11: HPLC spectrum of compound 26. Gradient: 20-60% CH_3CN/H_2O with 0.1% TFA over 30 min at a flow rate of 3.5 mL/min. Wavelength (nm):214nm.

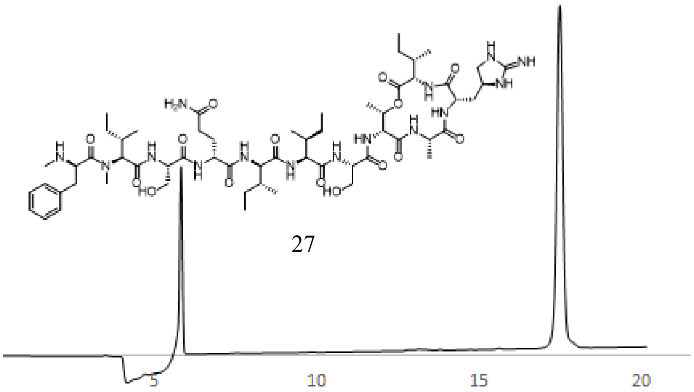

Supplementary Figure 12: HPLC spectrum of compound 27. Gradient: 20-60% CH_3CN/H_2O with 0.1% TFA over 30 min at a flow rate of 3.5 mL/min. Wavelength (nm):214nm.

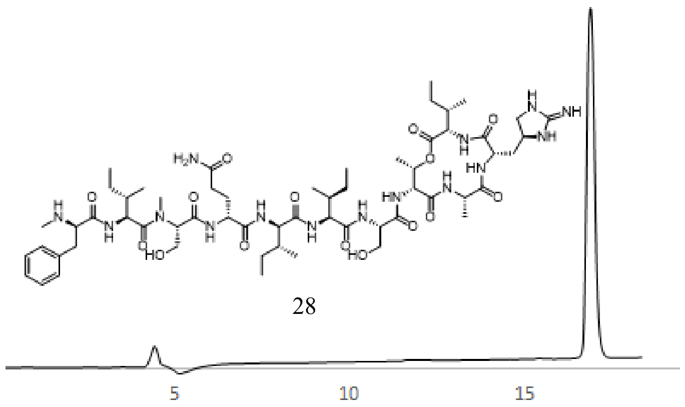

Supplementary Figure 13: HPLC spectrum of compound 28. Gradient: 20-60% CH_3CN/H_2O with 0.1% TFA over 30 min at a flow rate of 3.5 mL/min. Wavelength (nm):214nm.

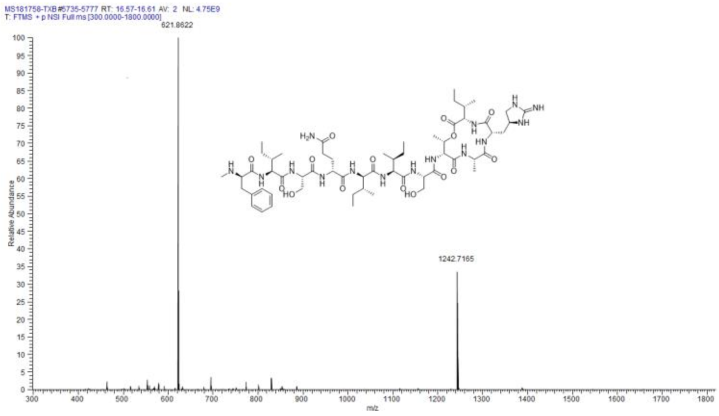

Supplementary Figure 14: HRMS spectrum of teixobactin (compound 19).

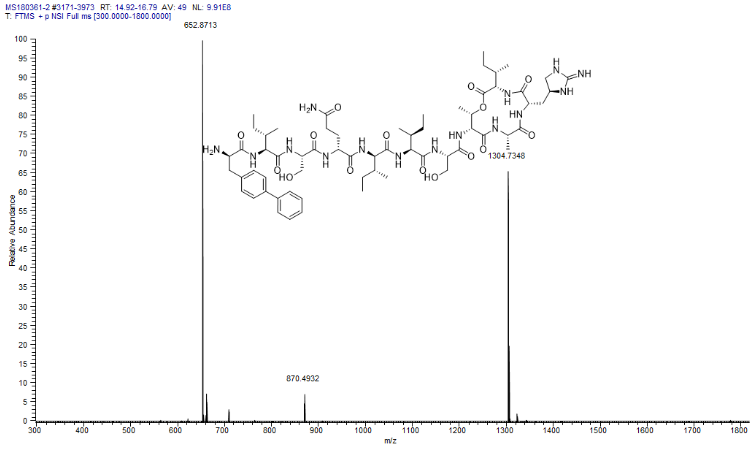

Supplementary Figure 15: HRMS spectrum of compound 20

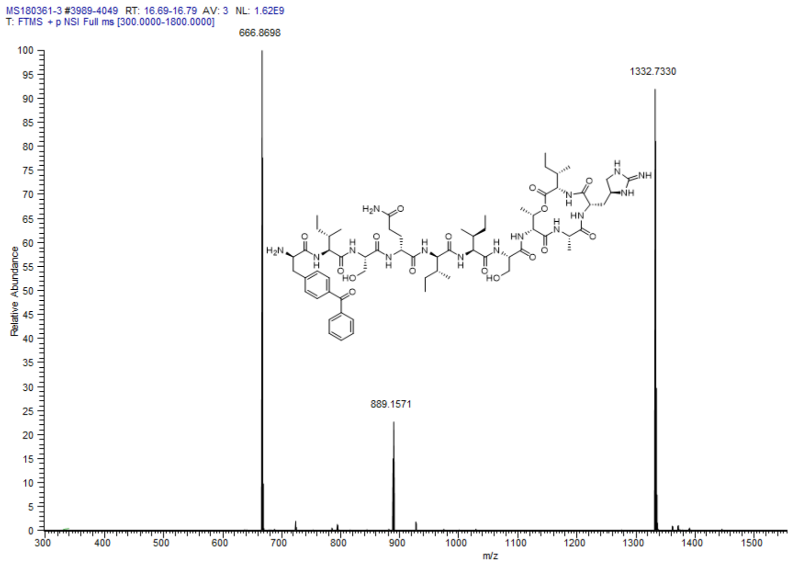

Supplementary Figure 16: HRMS spectrum of compound 21

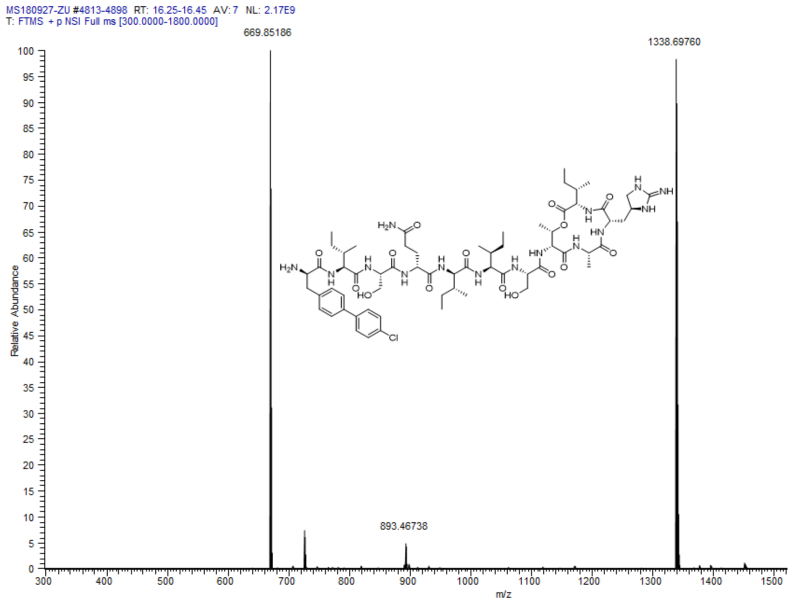

Supplementary Figure 17: HRMS spectrum of compound 22

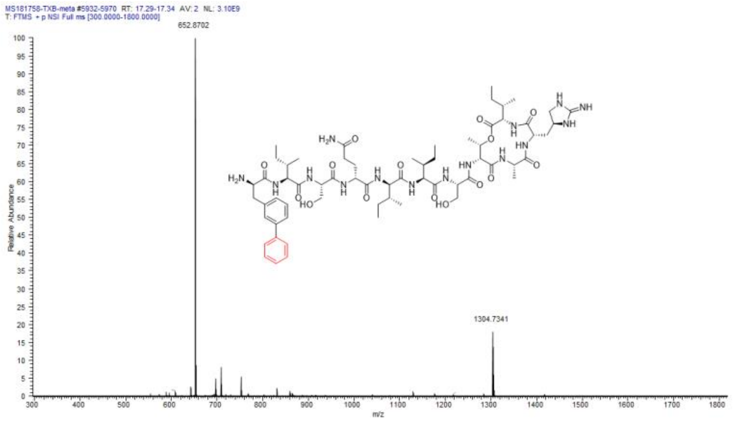

Supplementary Figure 18: HRMS spectrum of compound 23

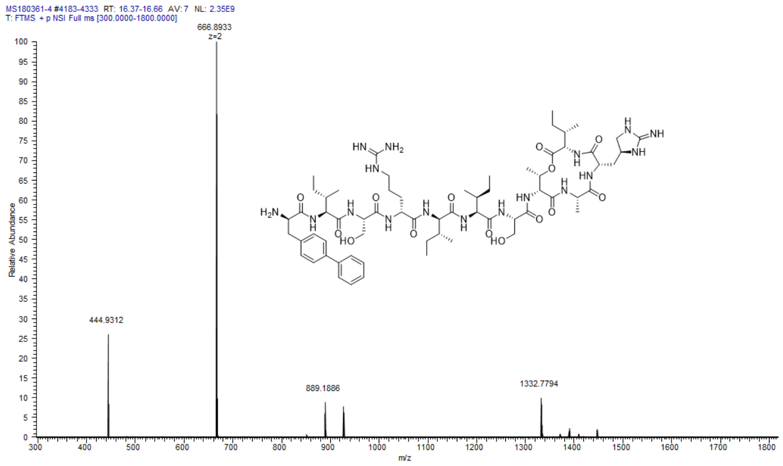

Supplementary Figure 19: HRMS spectrum of compound 24

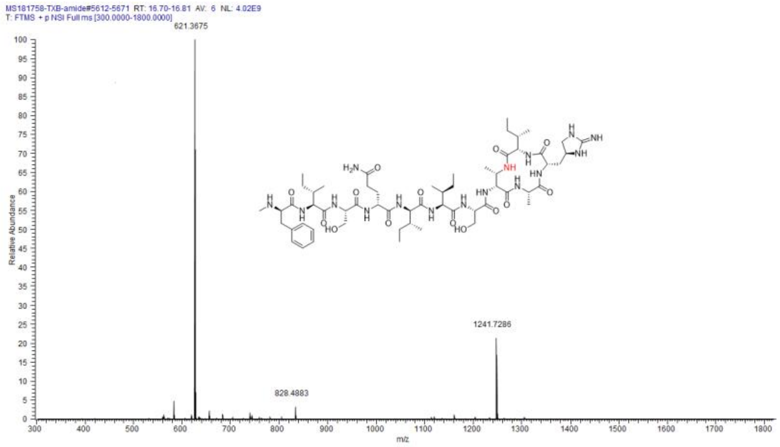

Supplementary Figure 20: HRMS spectrum of compound 25

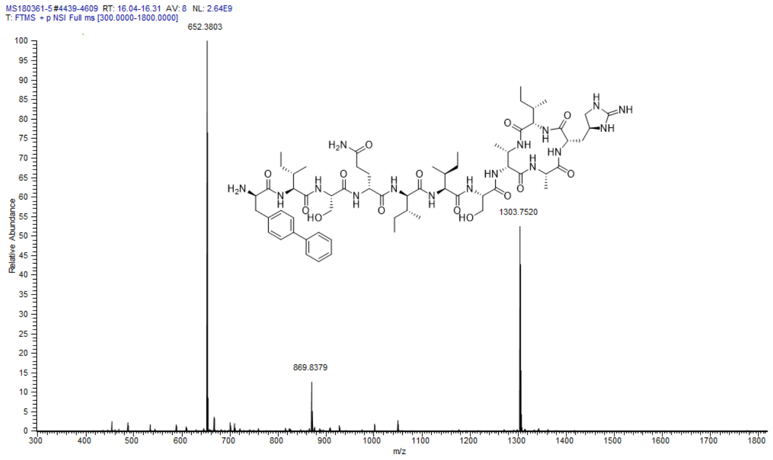

Supplementary Figure 21: HRMS spectrum of compound 26

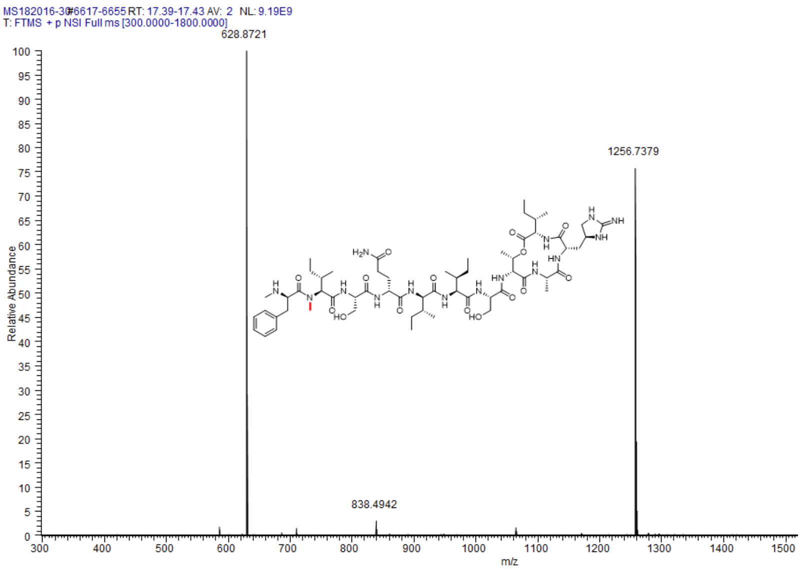

Supplementary Figure 22: HRMS spectrum of compound 27

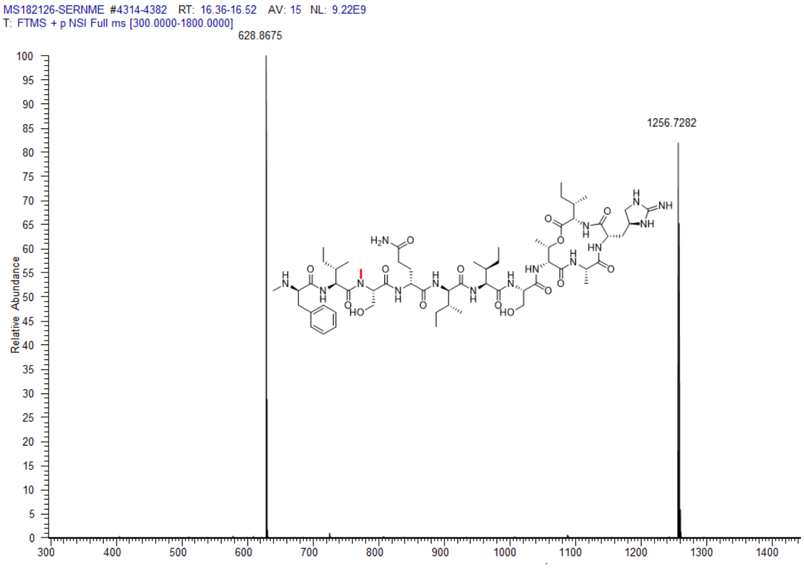

Supplementary Figure 23: HRMS spectrum of compound 28

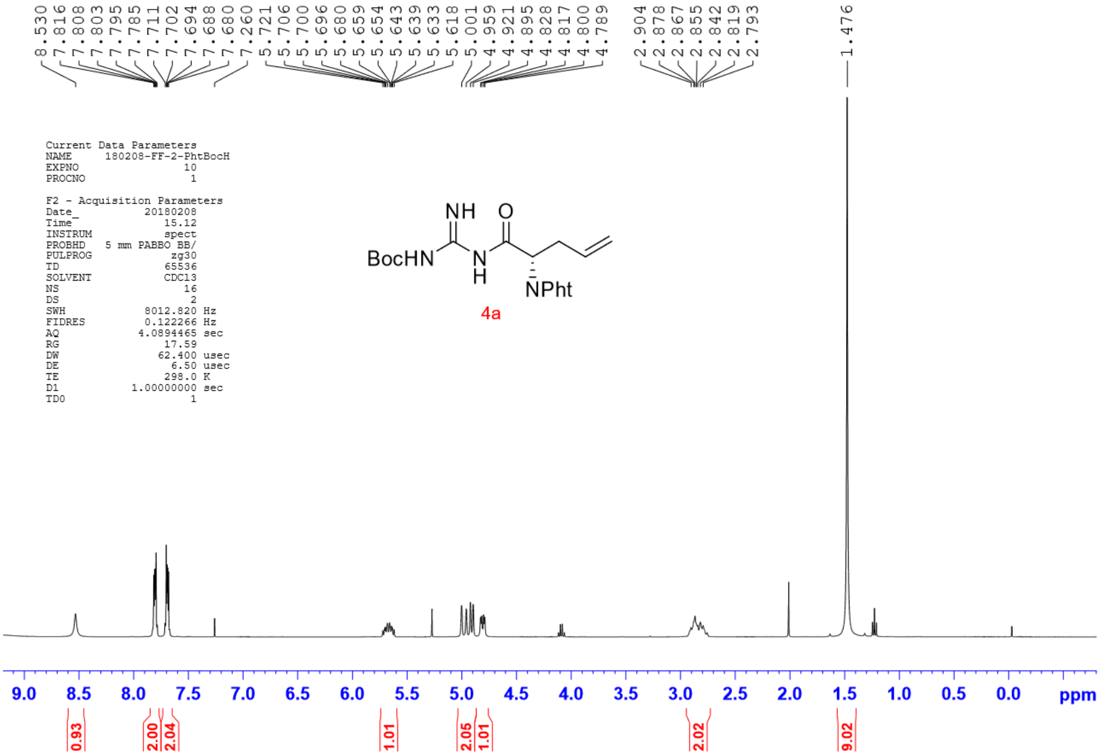

Supplementary Figure 24: ^{1}H NMR spectrum of compound 4a

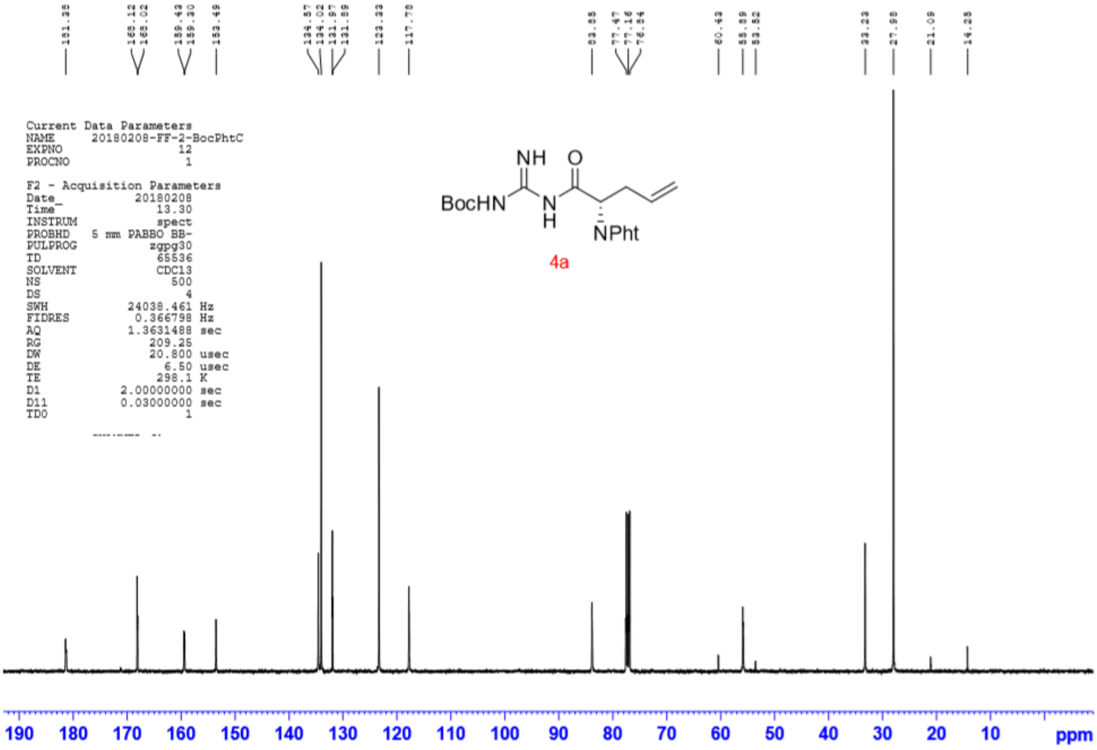

Supplementary Figure 25: ^{13}C NMR spectrum of compound 4a

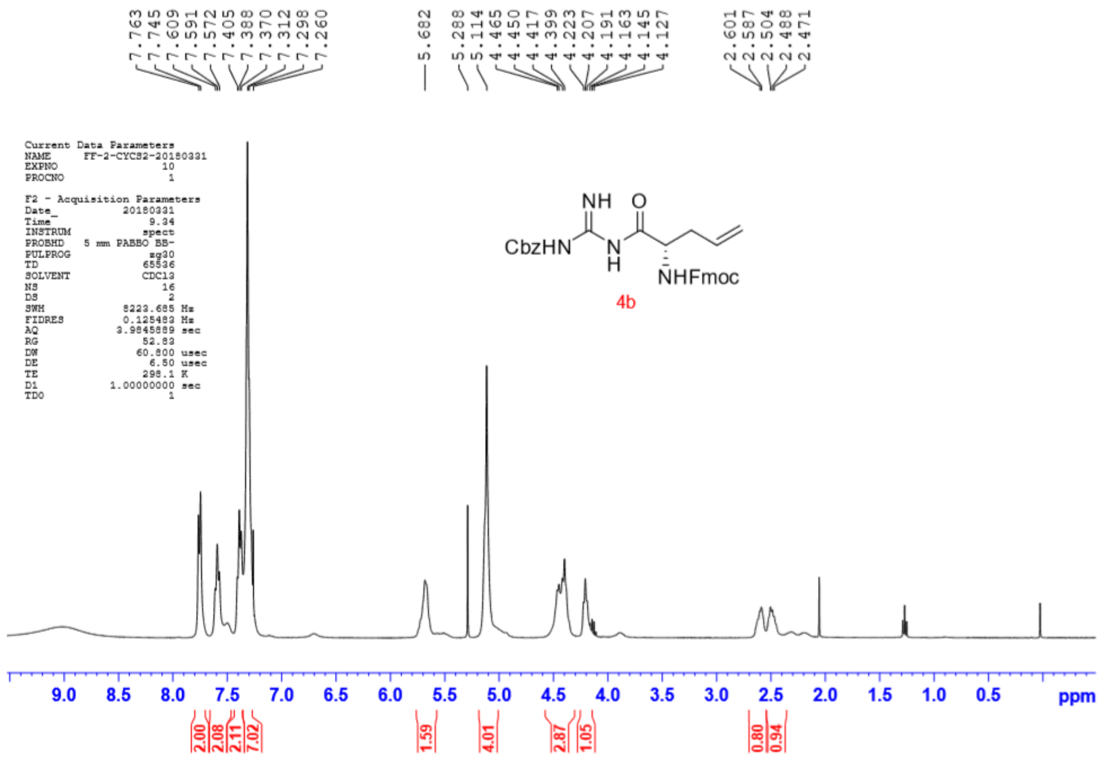

Supplementary Figure 26: ^{1}H NMR spectrum of compound 4b

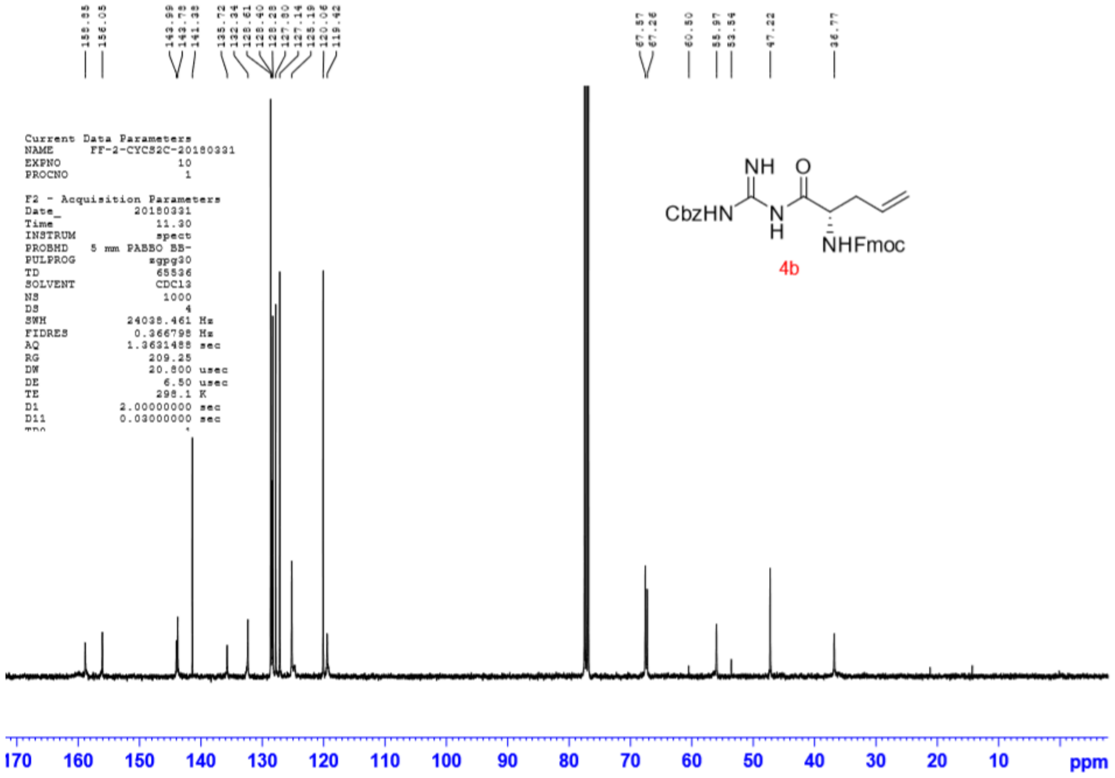

Supplementary Figure 27: ^{13}C NMR spectrum of compound 4b

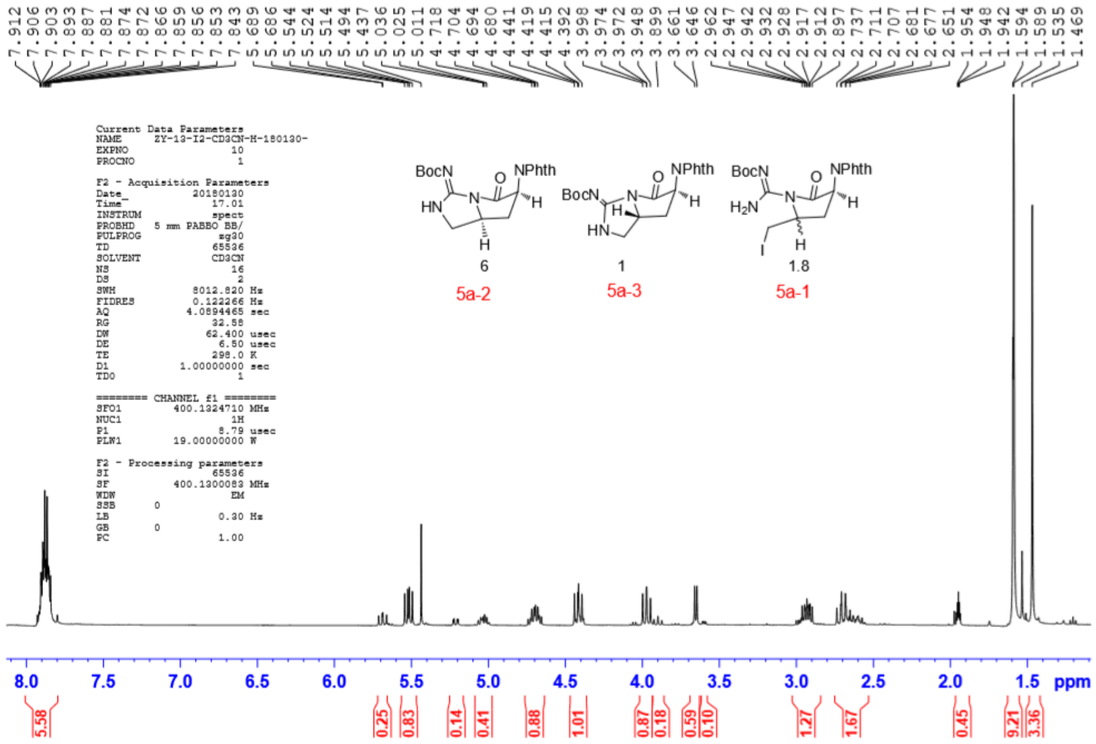

Supplementary Figure 28: ^{1}H NMR spectrum of compound 5a

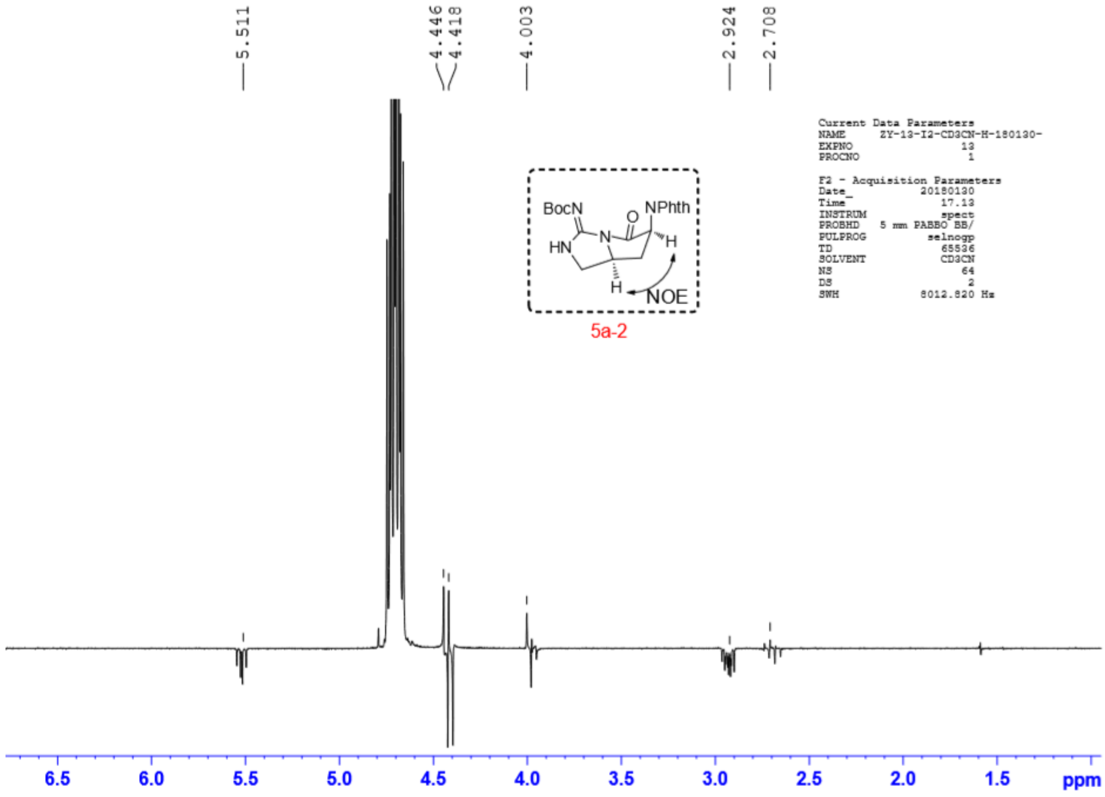

Supplementary Figure 29: NOE spectrum of compound 5a-2

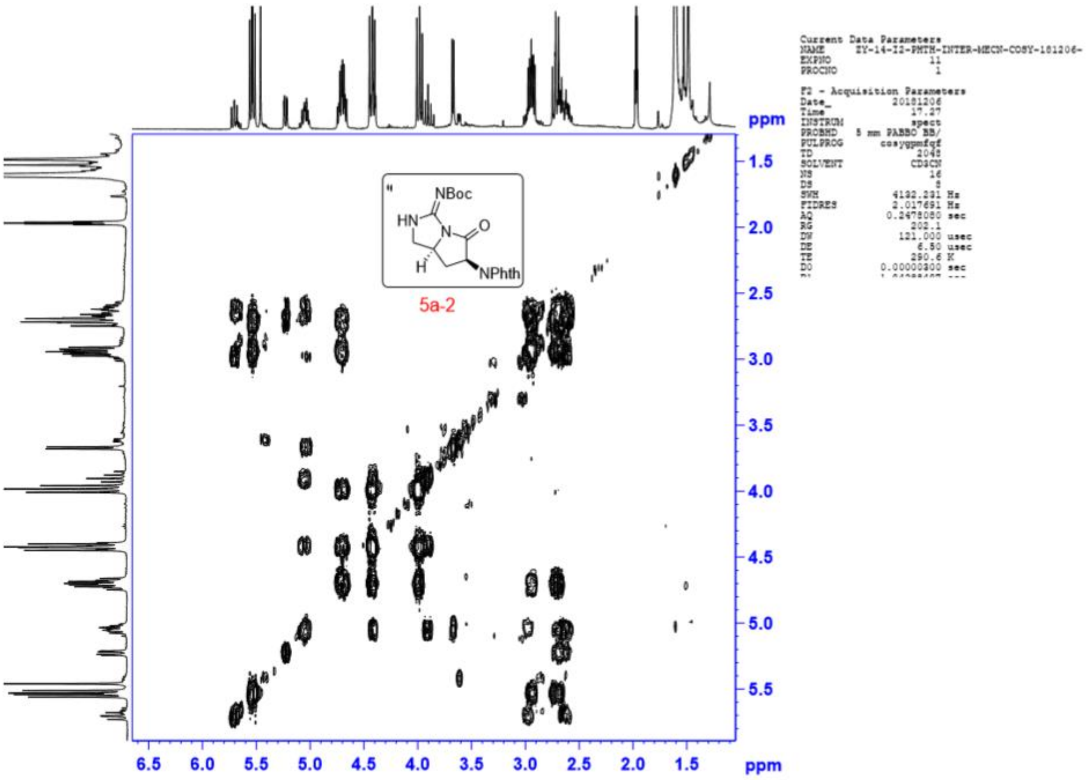

Supplementary Figure 30: COSY spectrum of compound 5a-2

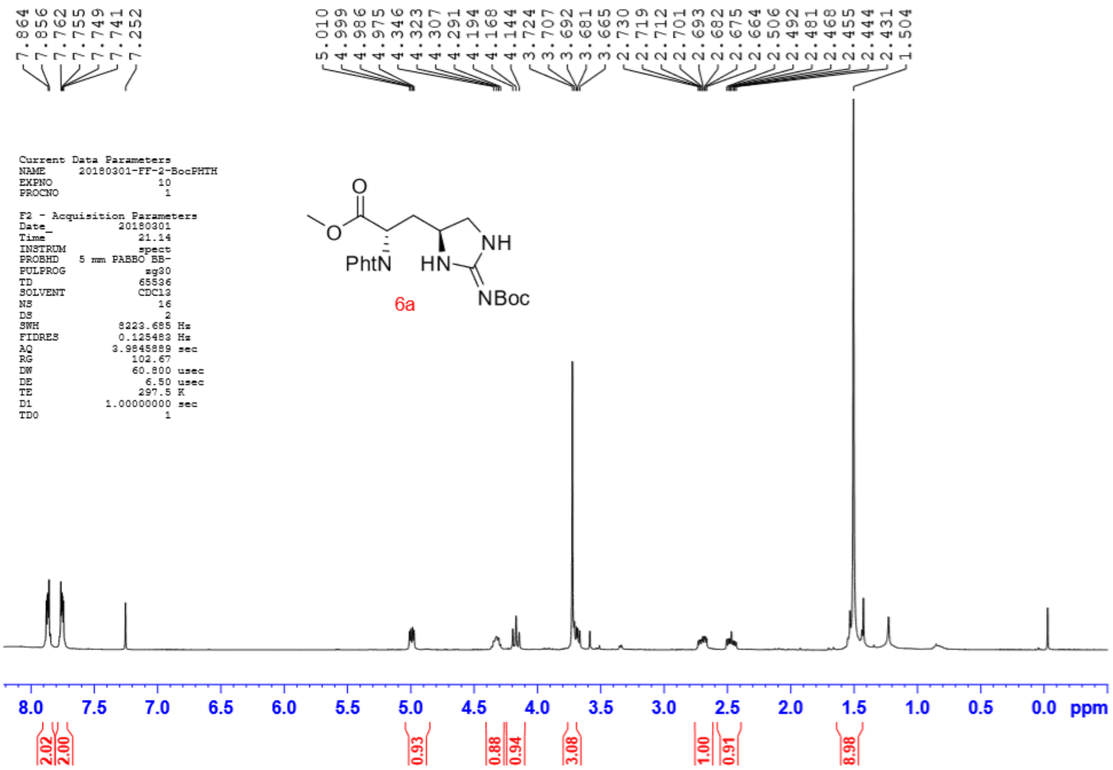

Supplementary Figure 31: ^{1}H NMR spectrum of compound 6a

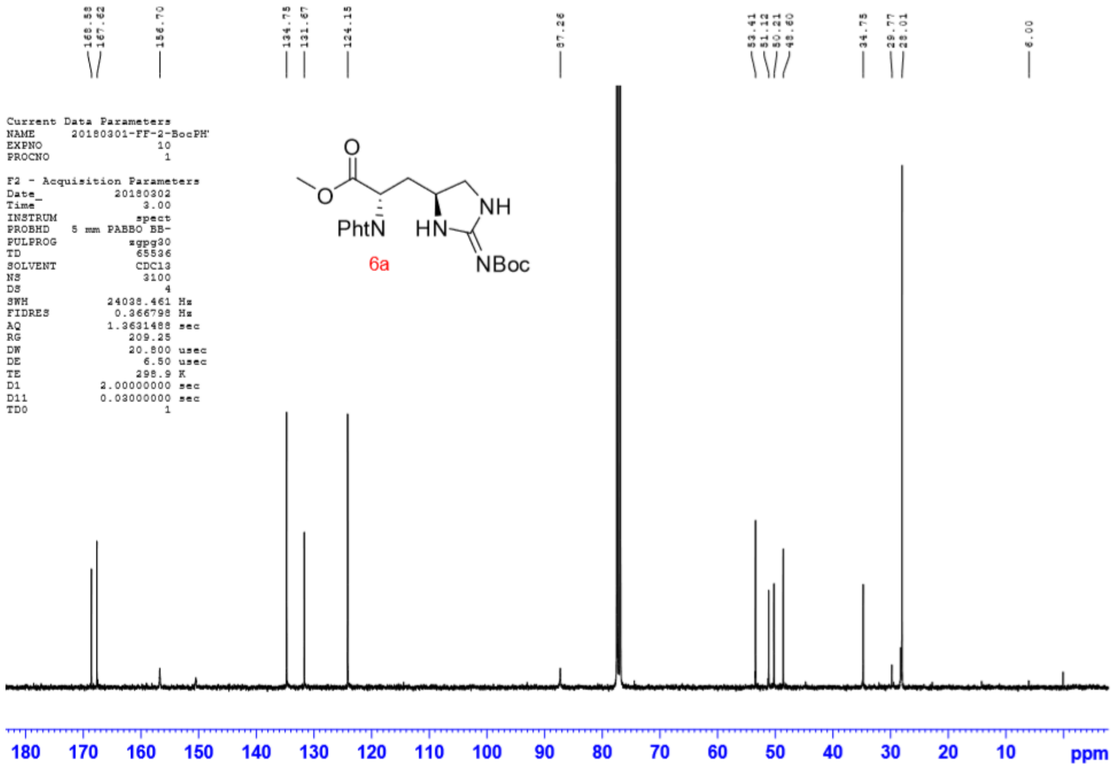

Supplementary Figure 32: ^{13}C NMR spectrum of compound 6a

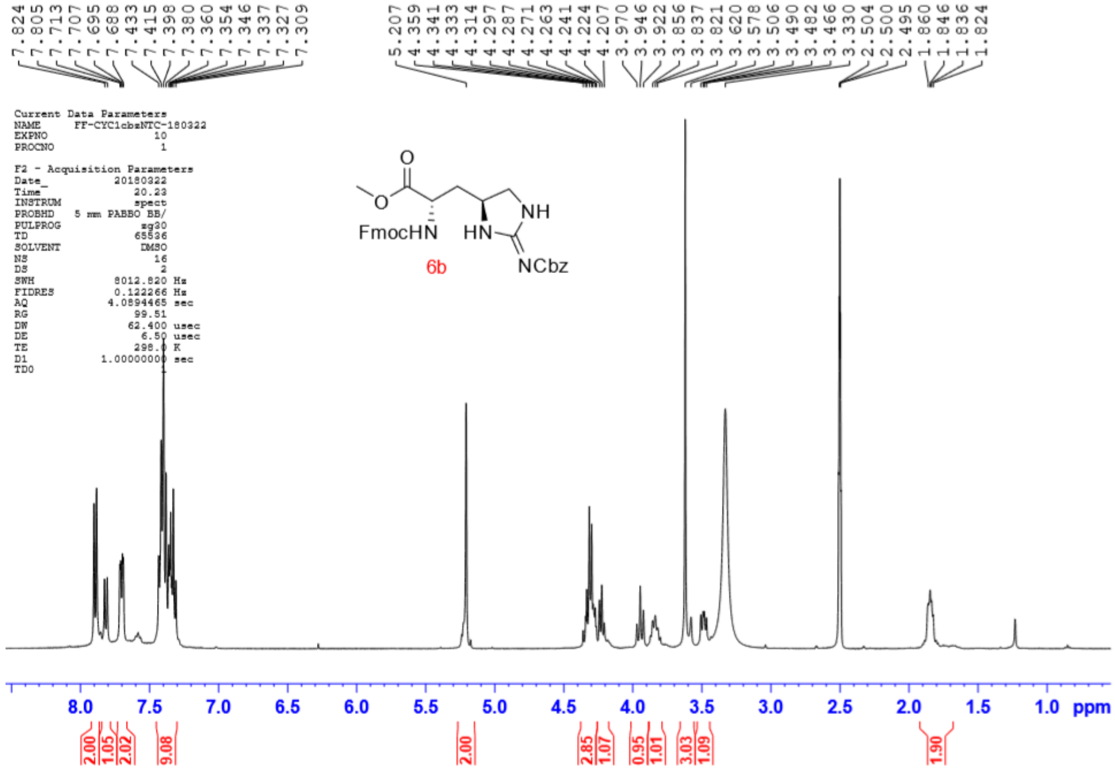

Supplementary Figure 33: ^{1}H NMR spectrum of compound 6b

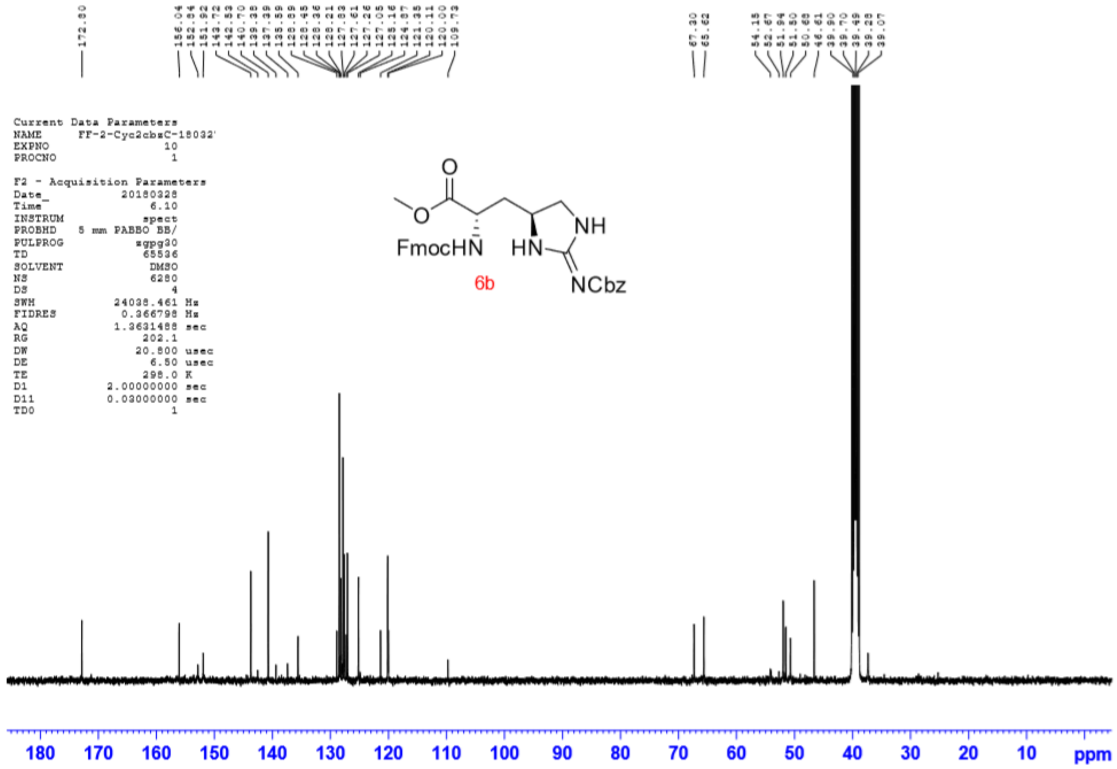

Supplementary Figure 34: ^{13}C NMR spectrum of compound 6b

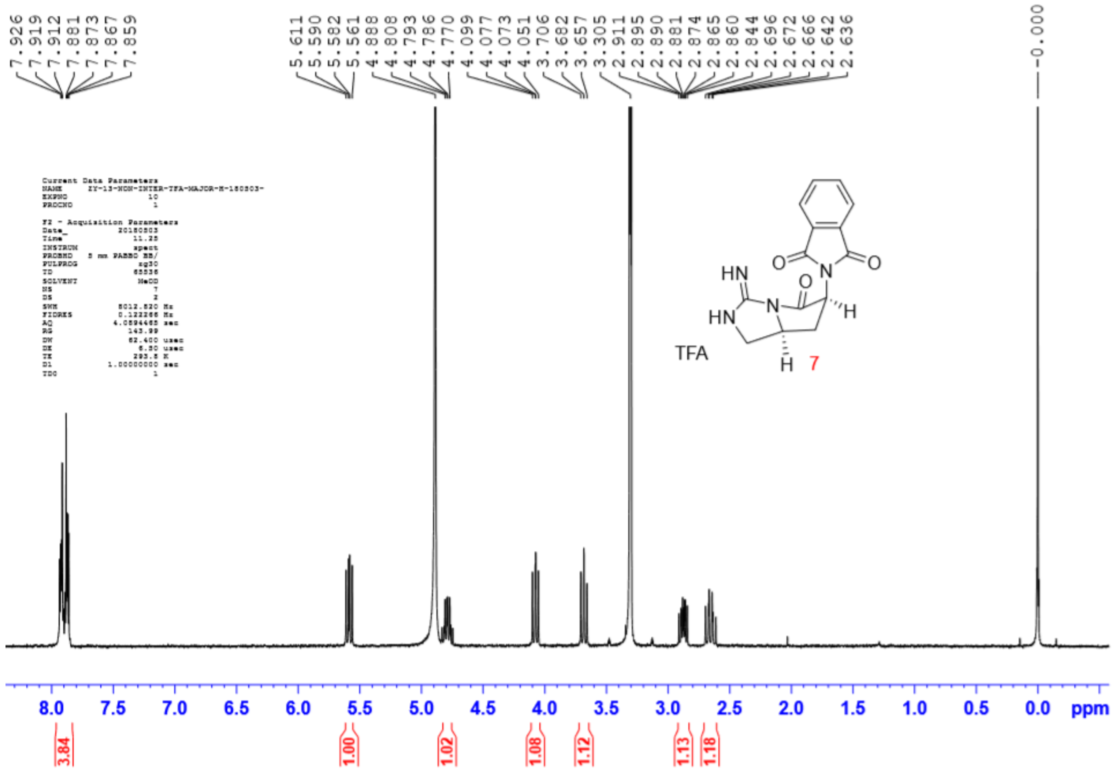

Supplementary Figure 35: ^{1}H NMR spectrum of compound 7

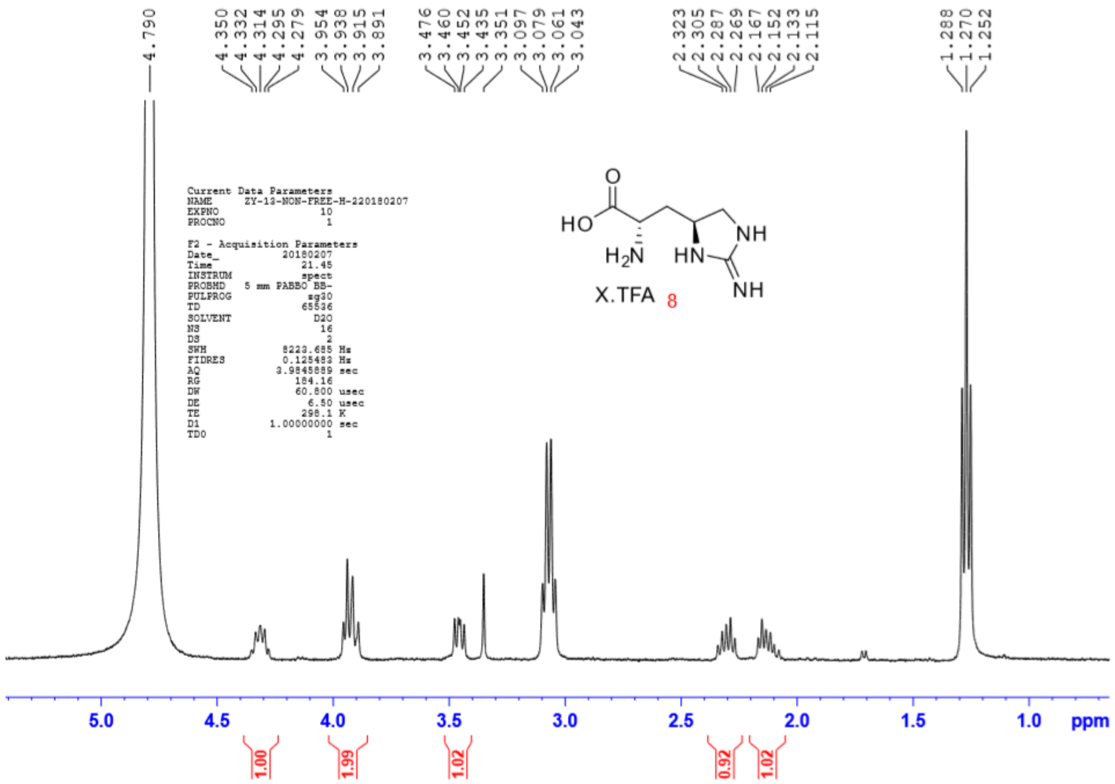

**Supplementary Figure 36: **

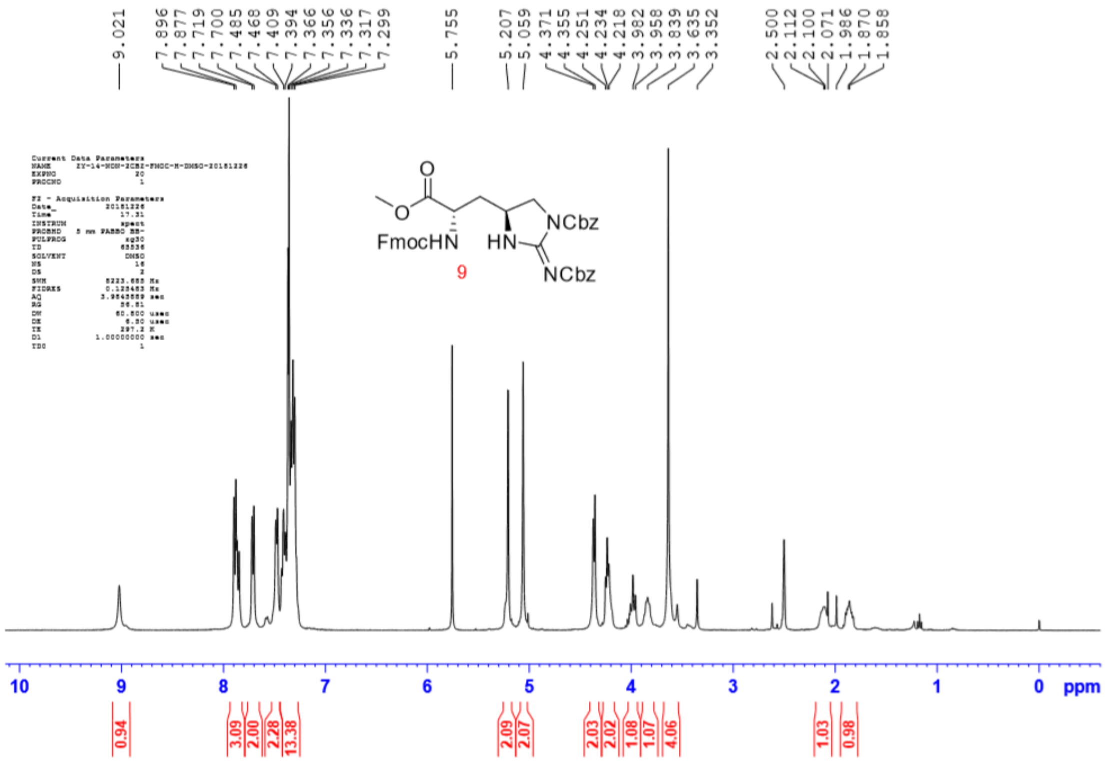

Supplementary Figure 37: ^{1}H NMR spectrum of compound 9

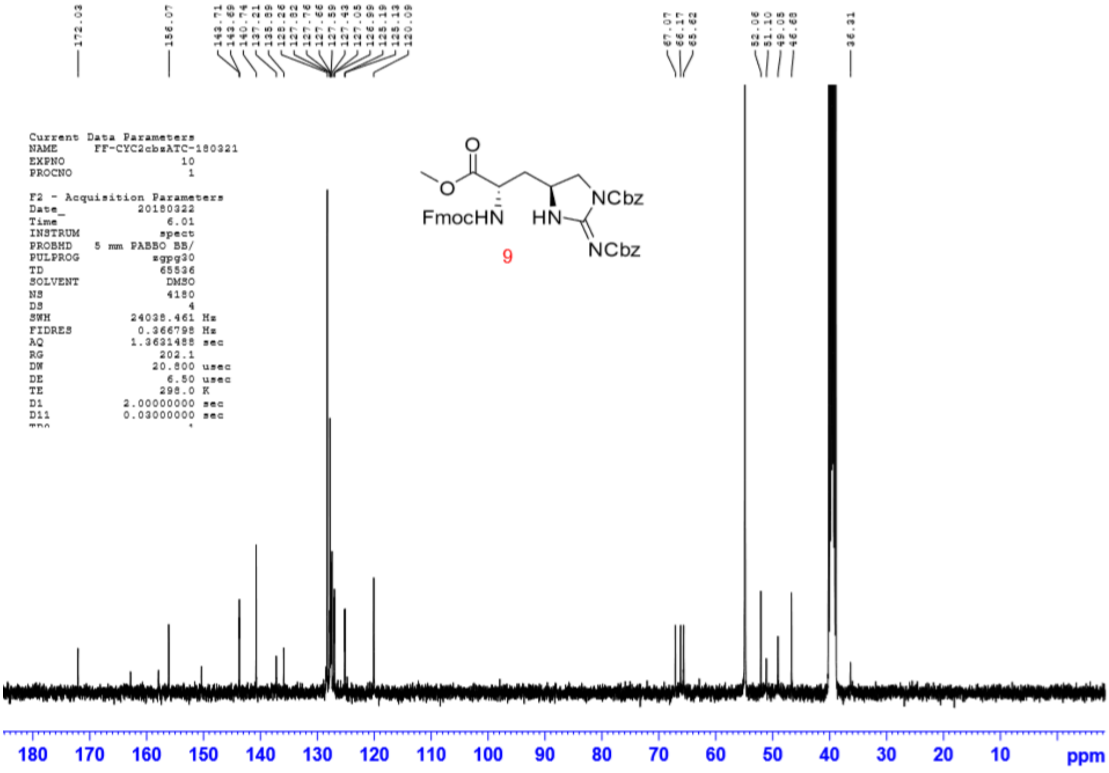

Supplementary Figure 38: ^{13}C NMR spectrum of compound 9

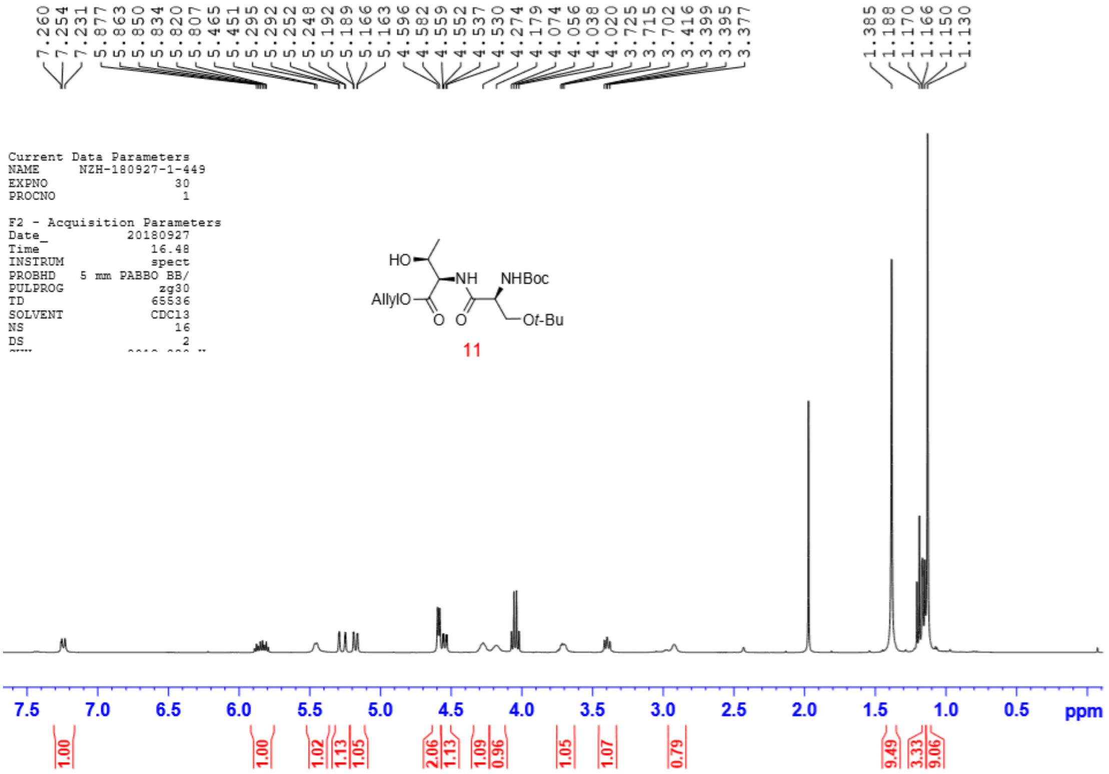

Supplementary Figure 39: ^{1}H NMR spectrum of compound 11

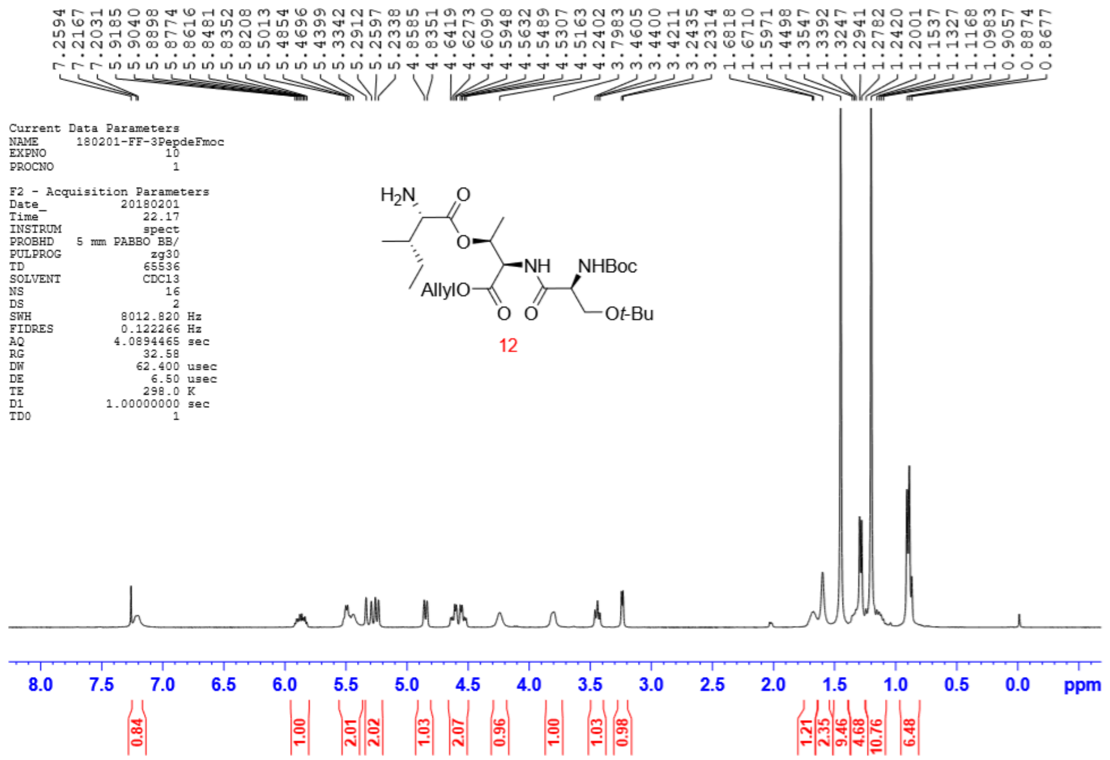

Supplementary Figure 40: ^{1}H NMR spectrum of compound 12

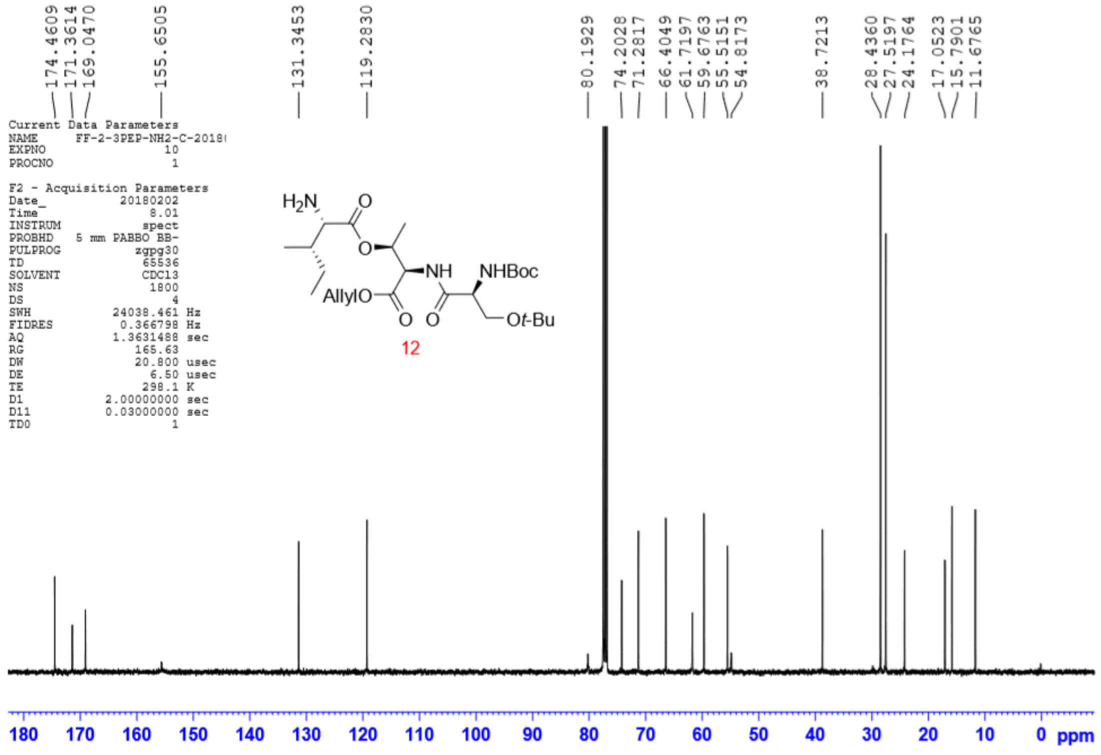

Supplementary Figure 41: ^{13}C NMR spectrum of compound 12

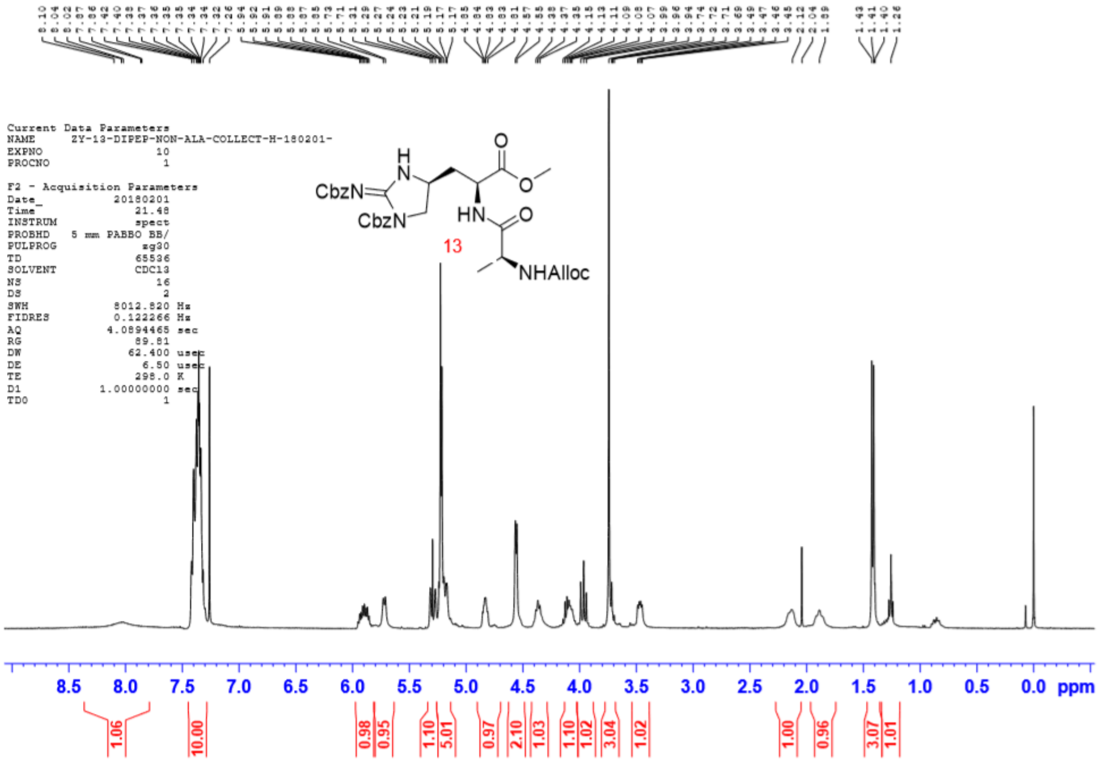

Supplementary Figure 42: ^{1}H NMR spectrum of compound 13

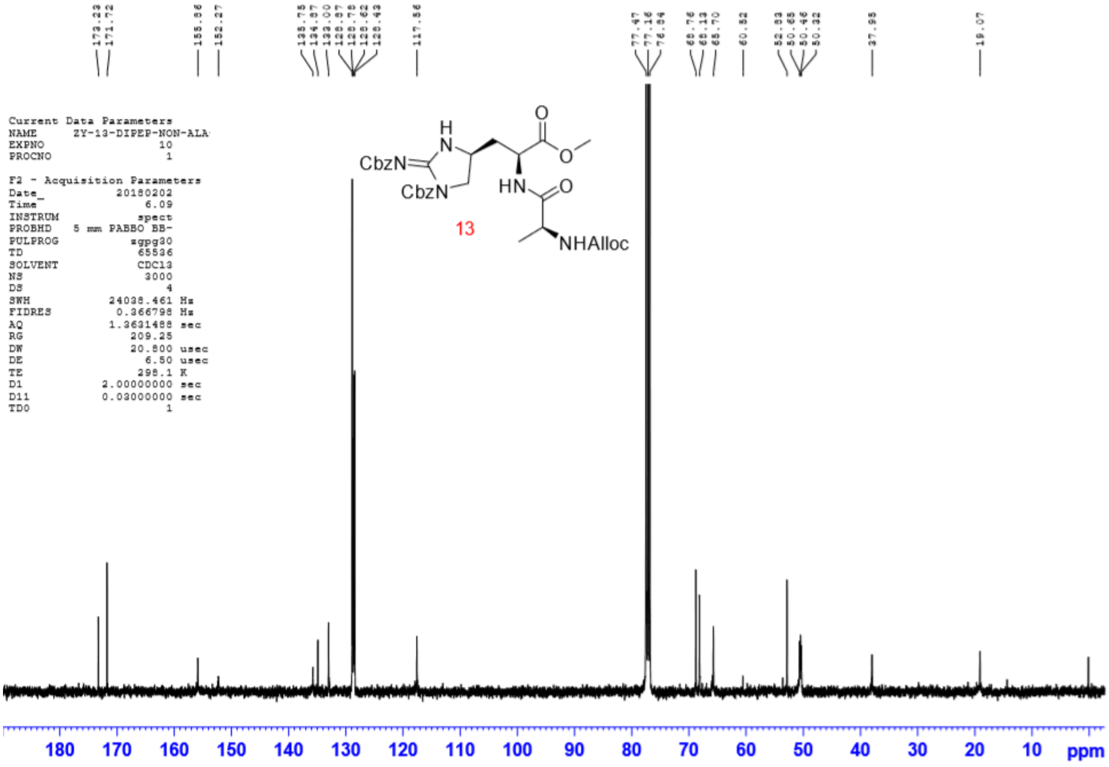

Supplementary Figure 43: ^{13}C NMR spectrum of compound 13

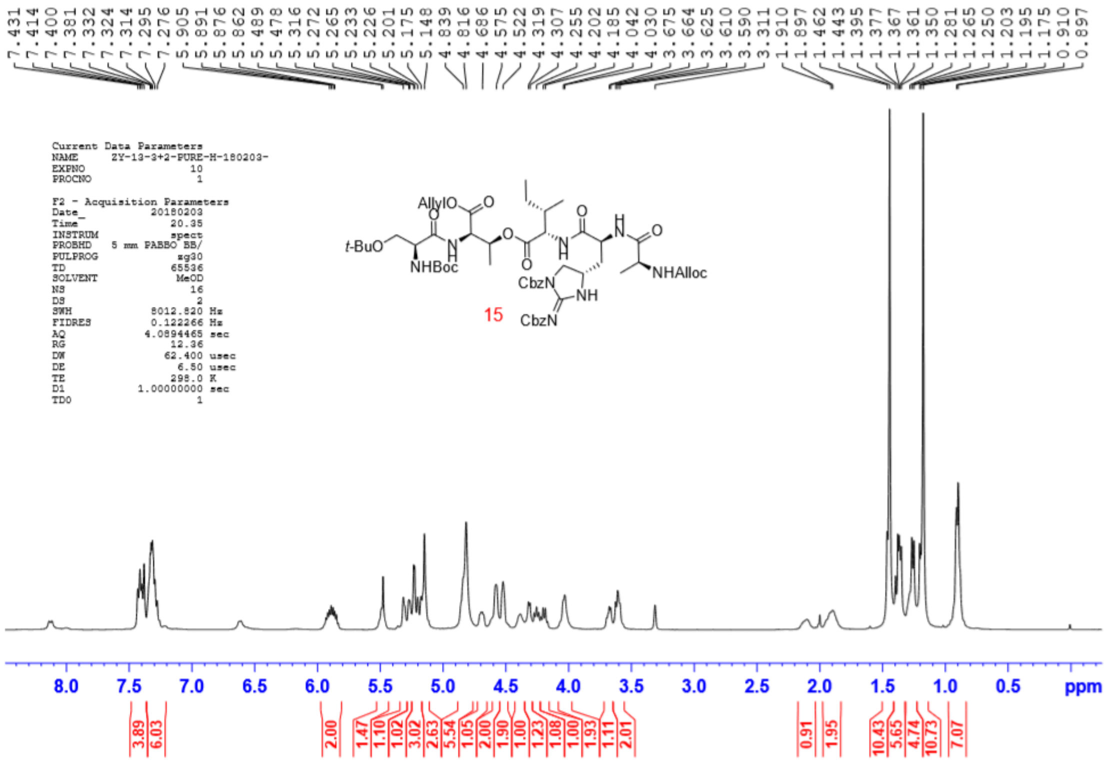

Supplementary Figure 44: ^{1}H NMR spectrum of compound 15

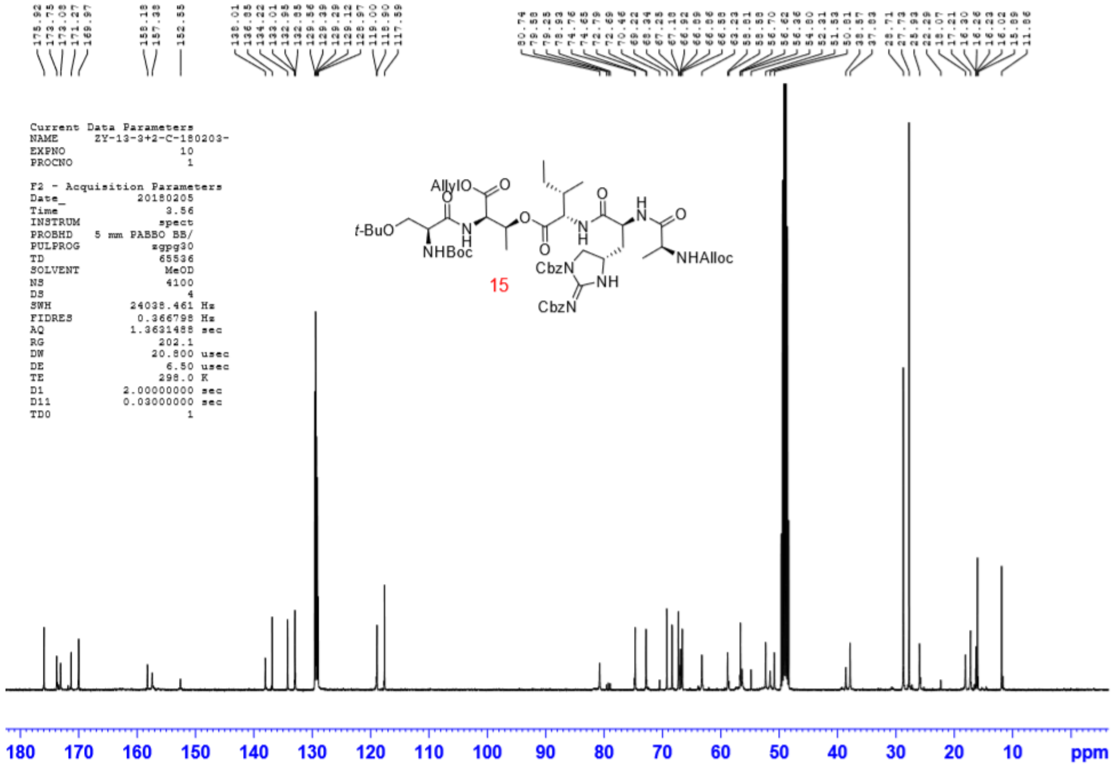

Supplementary Figure 45: ^{13}C NMR spectrum of compound 15

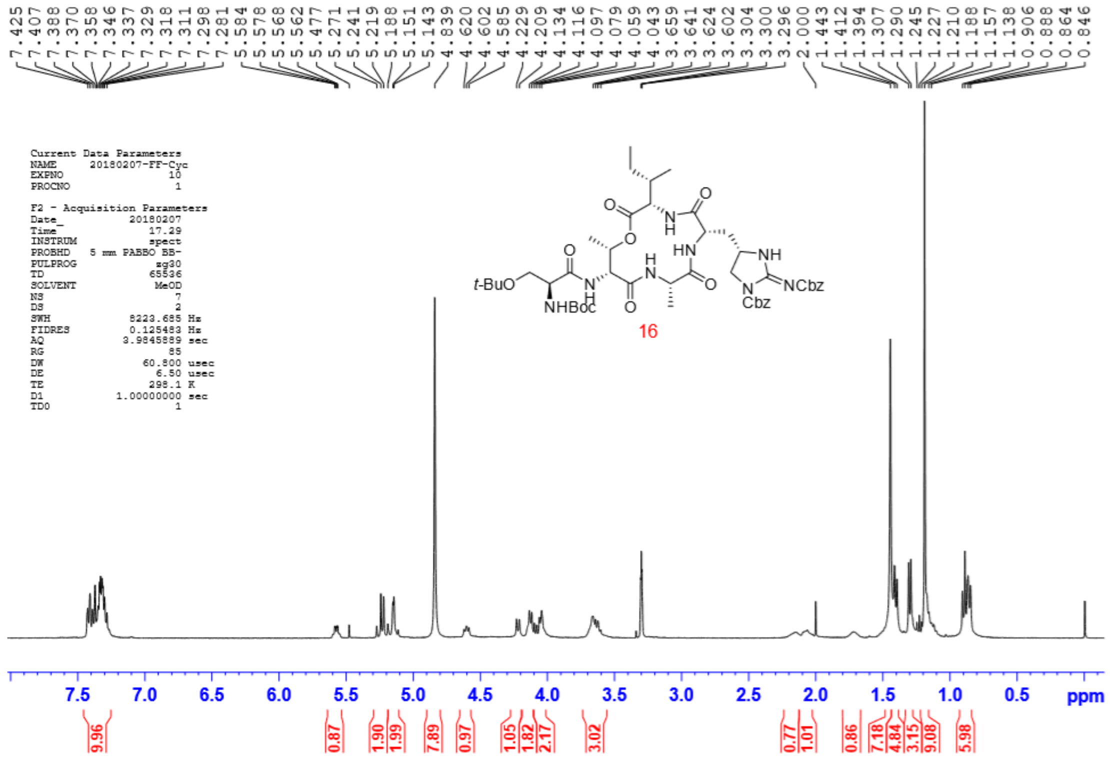

Supplementary Figure 46: **^{1}H NMR spectrum of compound 16**

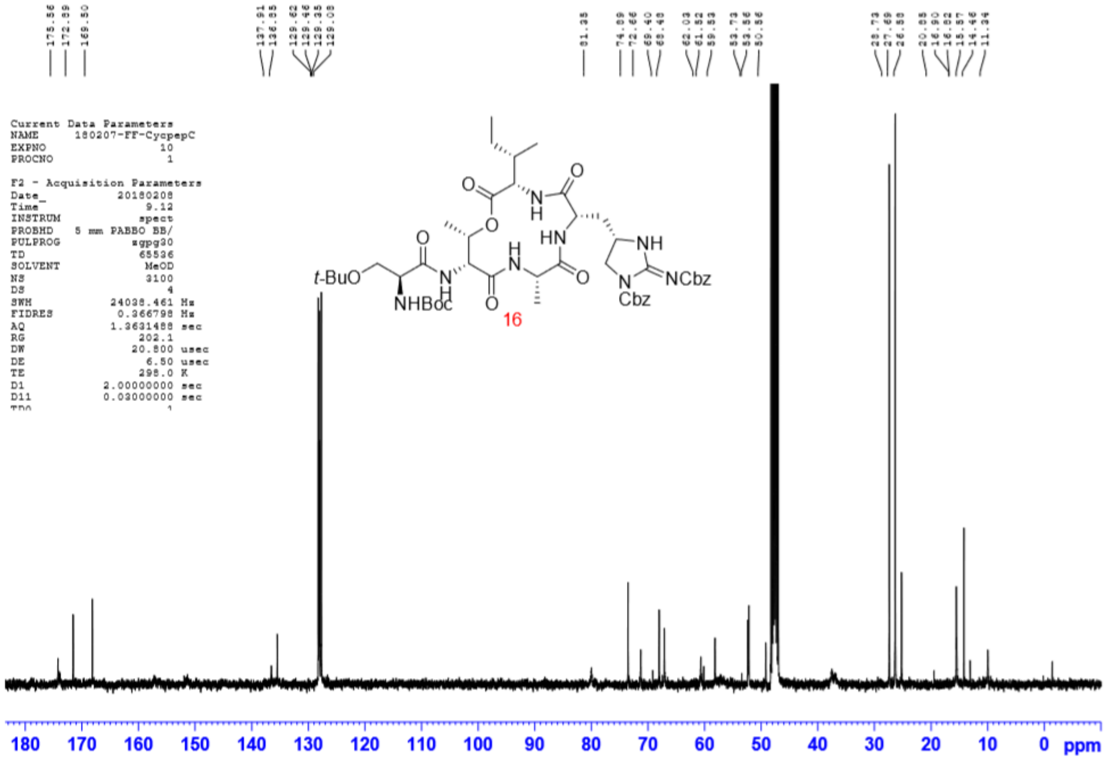

Supplementary Figure 47: ^{13}C NMR spectrum of compound 16

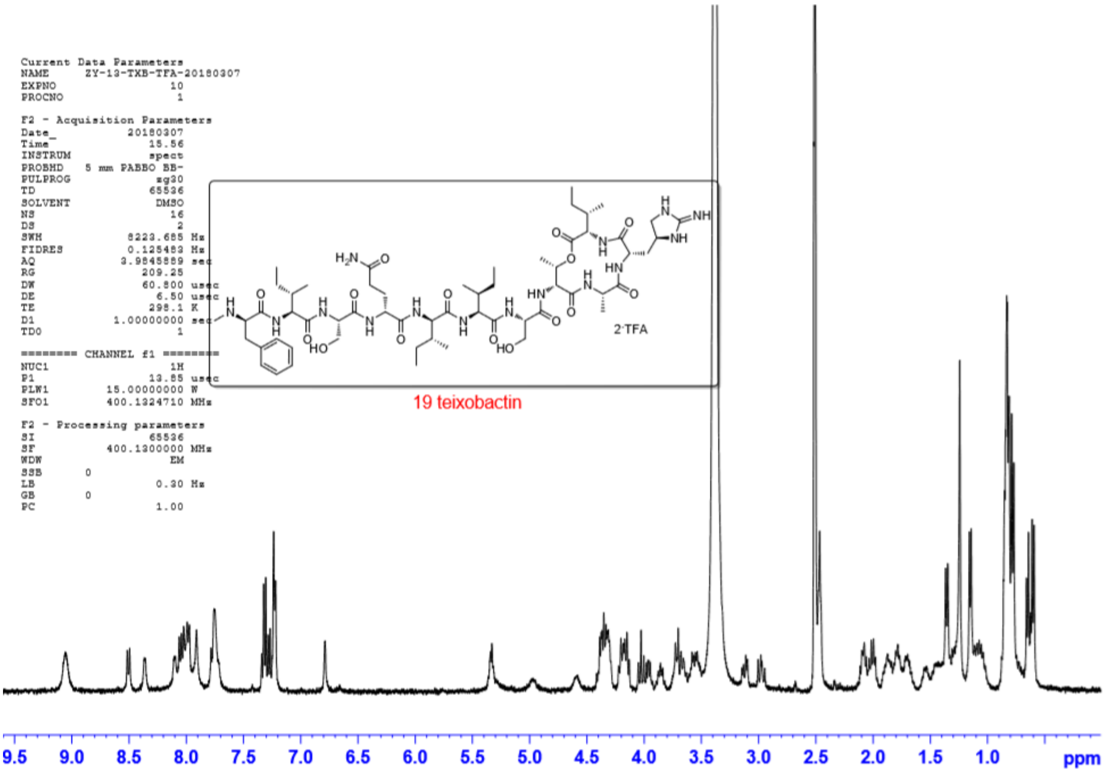

Supplementary Figure 48: ^{1}H NMR spectrum of compound 19 (TFA salt)

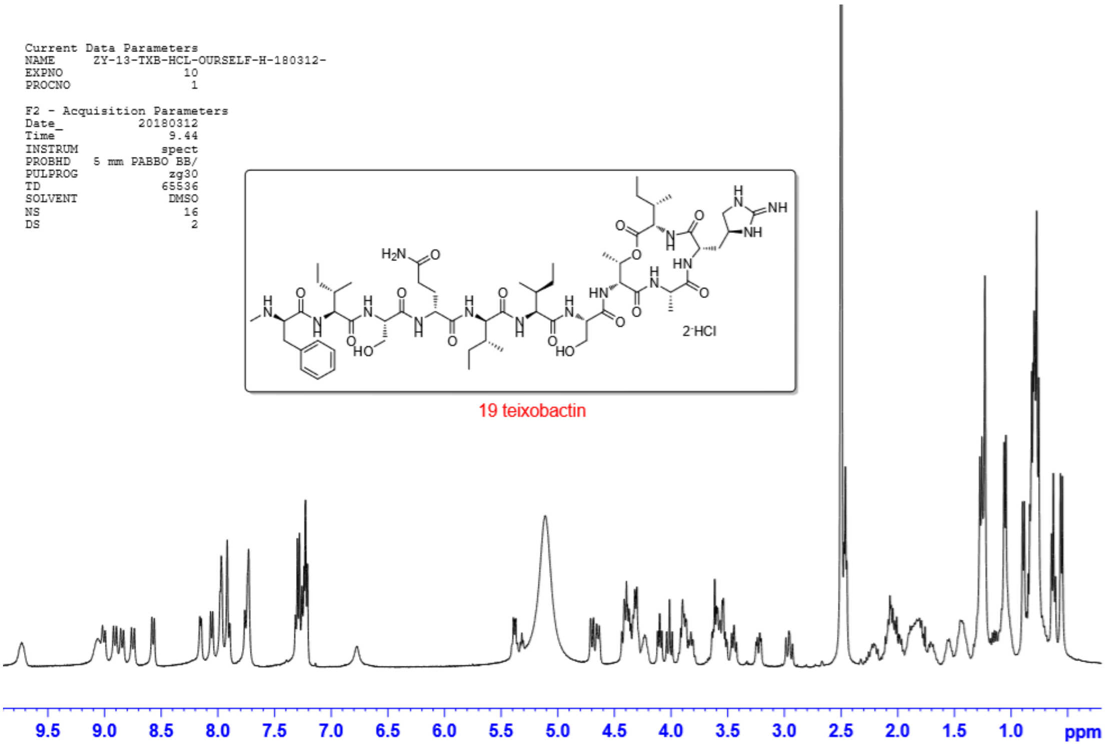

Supplementary Figure 49: ^{1}H NMR spectrum of compound 19 (HCl salt)

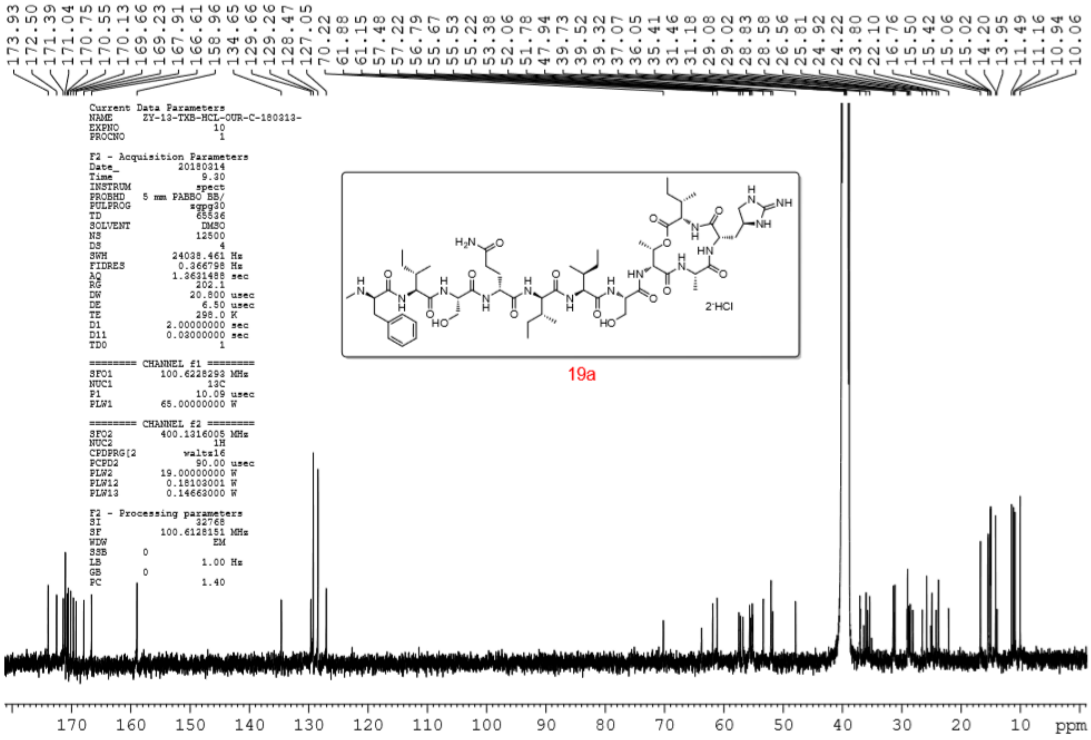

Supplementary Figure 50: ^{13}C NMR spectrum of compound 19 (HCl salt)

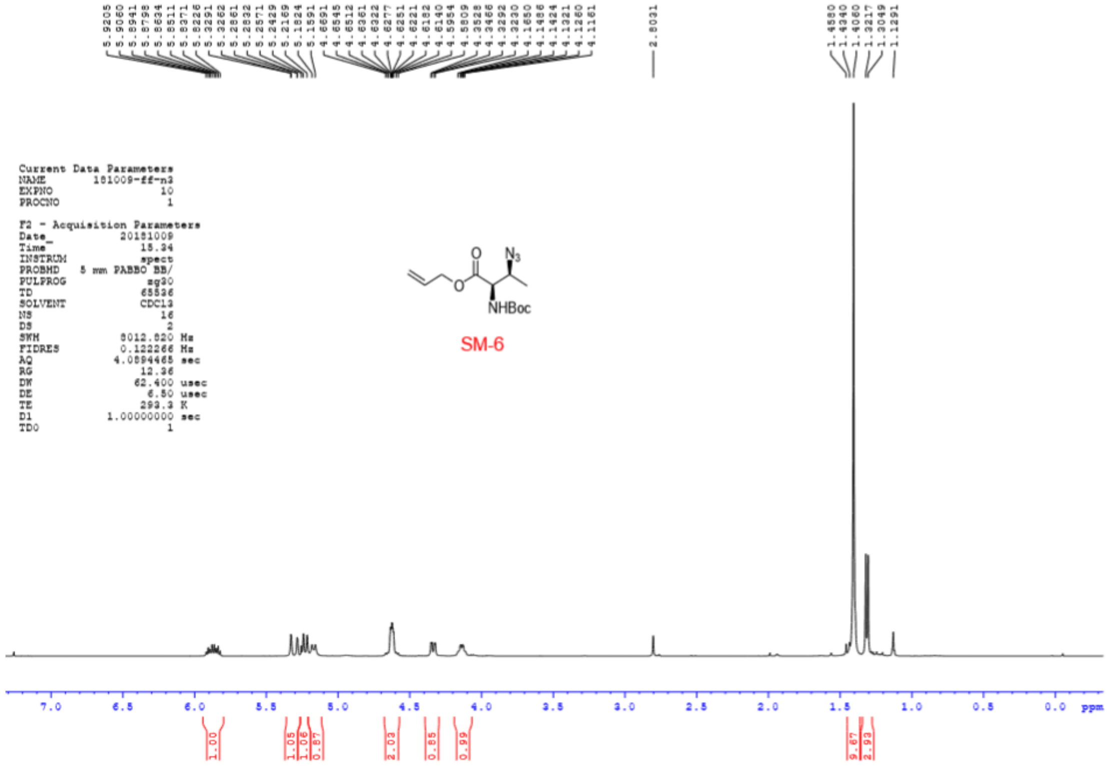

Supplementary Figure 51: ^{1}H NMR spectrum of compound SM-6

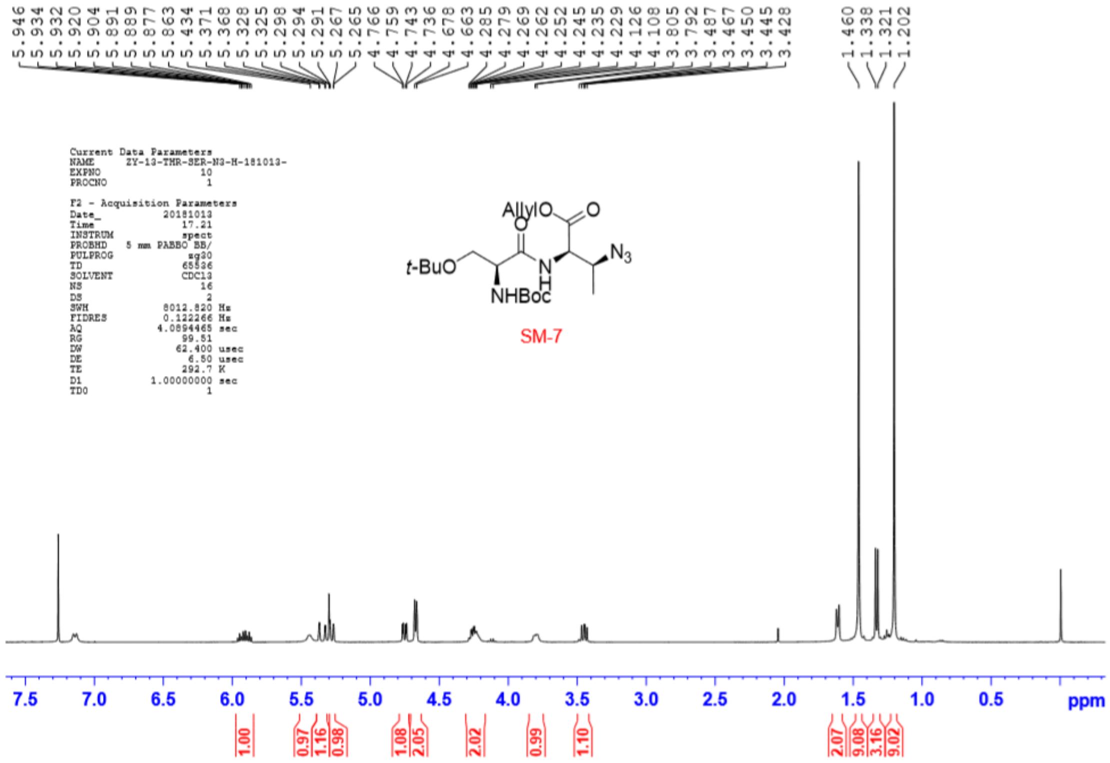

Supplementary Figure 52: ^{1}H NMR spectrum of compound SM-7

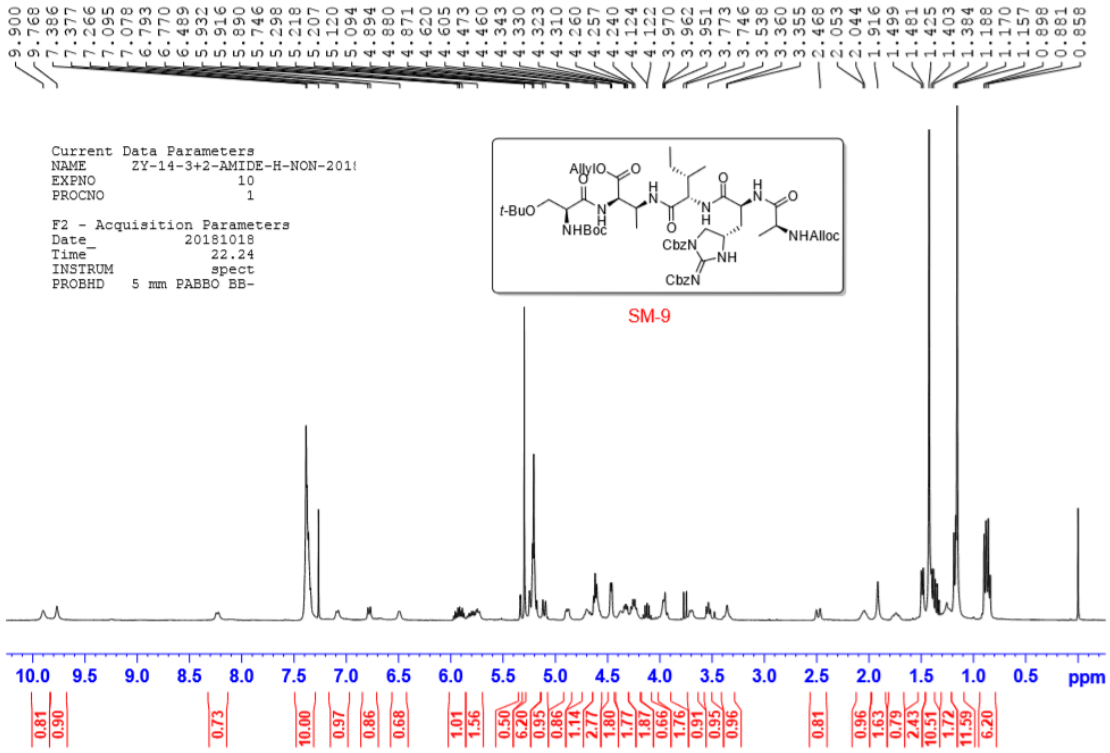

Supplementary Figure 53: ^{1}H NMR spectrum of compound SM-9

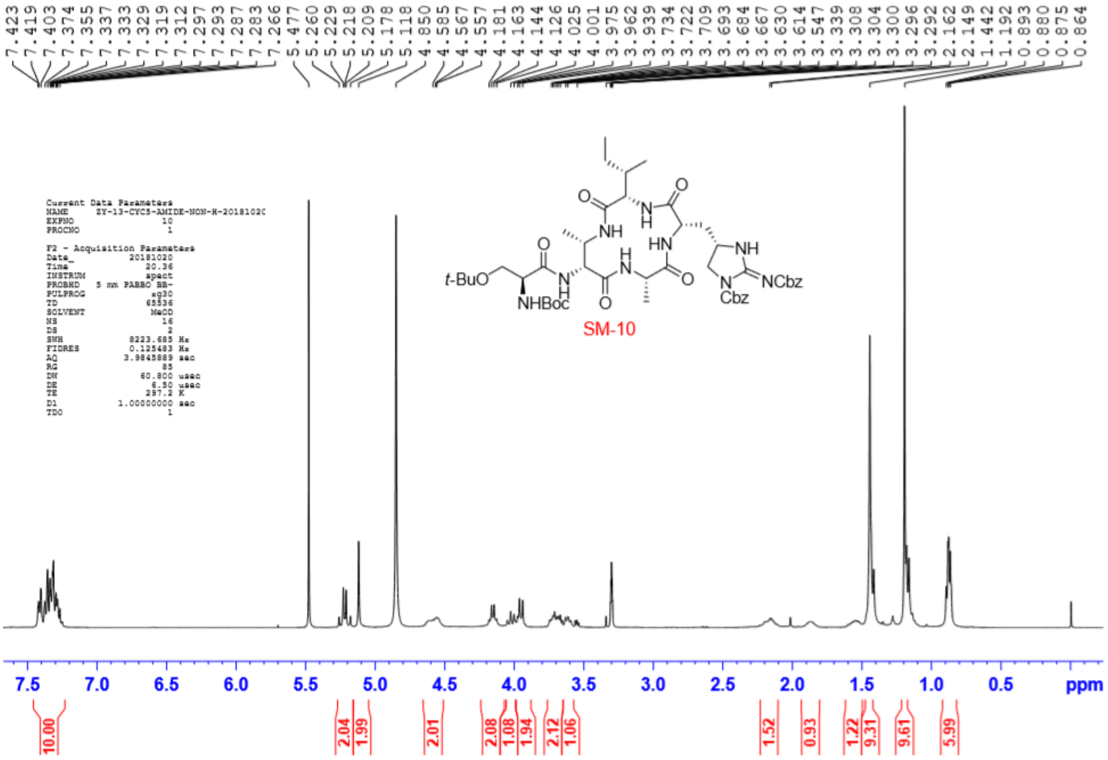

Supplementary Figure 54: ^{1}H NMR spectrum of compound SM-10

Supplementary Tables

Supplementary Table 1 MIC (μg ml^{-1}) for E. faecalis TH4938 and MRSA BAA-1695[a]

Strain	teixobactin	Compound **20**	**S-29**	**S-30**
E.faecalis TH4938	0.5	0.09	0.375	0.75
MRSA BAA-1695	0.25	0.125	0.5	0.5

a) It was done three times to confirm the results (n = 3). Source data are provided as a Source Data file.

Supplementary Table 2 Minimum bactericidal concentration (MBC) (μg ml^{-1}) for MRSA

Strain	teixobactin	Compound **20**
MRSA BAA-1695[a]	0.5	0.125
MRSA ATCC 33591[b]	0.28 +/- 0.14	0.12 +/- 0.08

a)It was done three times to confirm the results (n = 3). b) Data represents 5 parallel experiments ± s.d. It was conducted five times to confirm the results (n = 5). Source data are provided as a Source Data file.

Supplementary Table 3 CD spectrum of teixobactin and compound 27

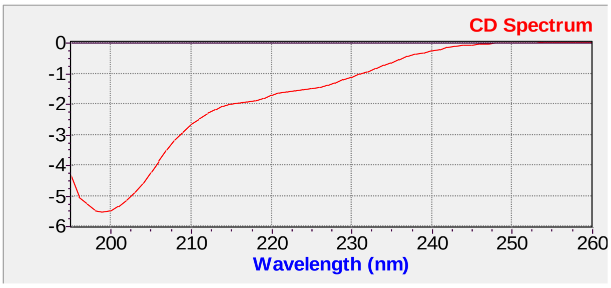

teixobactin	195-260 nm	200-260 nm	205-260 nm	210-260 nm
Helix	10.50%	11.90%	13.40%	15.70%
**Antiparallel**	**29.70%**	**37.40%**	**34.90%**	**30.00%**
Parallel	4.40%	4.90%	5.10%	5.40%
Beta-Turn	20.50%	21.80%	21.40%	20.00%
Rndm. Coil	37.50%	33.40%	30.90%	33.60%
Total Sum	102.60%	109.40%	105.90%	104.80%

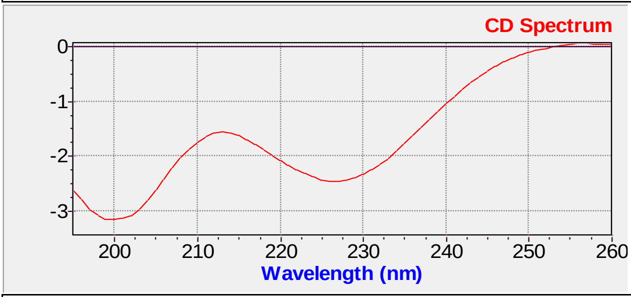

Compound **27**	195-260 nm	200-260 nm	205-260 nm	210-260 nm
Helix	18.00%	22.10%	24.90%	26.90%
**Antiparallel**	**14.60%**	**18.70%**	**16.60%**	**15.00%**
Parallel	5.80%	6.10%	5.90%	5.80%
Beta-Turn	16.40%	17.60%	17.30%	17.30%
Rndm. Coil	47.30%	40.00%	36.10%	35.90%
Total Sum	102.10%	104.40%	100.70%	100.90%

Supplementary Table 4 ^{1}H NMR data of teixobactin

entry	Compound **19**	teixobactin[a]	entry	Compound **19**	teixobactin[a]
1	2.47 (3H, brs)	2.47 (3H, brs)	29	4.37 (1H, m)	4.37 (1H, m)
2	4.23 (1H, m)	4.23 (1H, m)	29-NH	7.90 (1H, m)	7.90 (1H ,m)
2-NH	9.73, 9.06 (2H, v br s)	9.66, 9.05 (2H, v br s)	30	1.83 (1H, m)	1.83 (1H, m)
3	2.96 (1H, dd， 13, 10.3)	2.96 (1H,dd,13.04, 10.2)	31	0.89 (3H, d, 6.68)	0.89 (3H, d, 6.6)
	3.23 (1H, dd,13.12, 4.8)	3.22 (1H, dd, 12.76, 4.8)	32	1.07 (1H, m)	1.07 (1H, m)
				1.44 (1H, m)	1.44 (1H, m)
5,5’	7.23 (2H, m)	7.23 (2H, m)	33	0.77 (1H, m)	0.77 (1H, m)
6,6’	7.30 (2H, m)	7.30 (2H, m)			
7	7.26 (1H, m)	7.26 (1H, m)	35	4.64 (1H, m)	4.64 (1H, m)
			35-NH	9.01 (1H, d, 9.28)	9.01 (1H, d, 9.59)
9	4.10 (1H, t, 7.36)	4.10 (1H, t, 7.68)	36	3.56 (1H, m)	3.56 (1H, m)
9-NH	8.56 (1H, d, 8.48)	8.57 (1H, d, 8.08)		3.87 (1H, m)	3.87 (1H, m)
10	1.55 (1H, m)	1.55 (1H, m)	37		
11	0.55 (3H, d, 6.76)	0.55 (3H, d, 6.72)	38	4.70 (1H, d, 11.6)	4.71 (1H, d, 11.32)
12	0.74 (1H, m)	0.74 (1H, m)	38-NH	8.91 (1H, d, 10.08)	8.92 (1H, d, 9.92)
	1.06 (1H, m)	1.06 (1H, m)	39	5.38 (1H, m)	5.38 (1H, m)
			40	1.05 (3H, d, 6.32)	10.5 (3H, d, 6.32)
13	0.63 (3H, t, 6.96)	0.63 (3H, t, 7.00)			
			42	3.89 (1H, m)	3.89 (1H, m)
15	4.32 (1H, m)	4.32 (1H, m)	43	1.26 (3H, d, 7.32)	1.26 (3H, d, 7.36)
15-NH	8.06 (1H, d, 7.76)	8.06 (1H, d, 7.92)			
16	3.53 (1H, m)	3.53 (1H, m)	45	4.37 (1H, m)	4.37 (1H, m)
	3.62 (1H, m)	3.62 (1H, m)	45-NH	8.85 (1H, d, 10.2)	8.85 (1H, d, 10.28)
16-OH	exchange		46	2.05(2H, m)	2.05(2H, m)
			47	3.82 (1H, m)	3.82 (1H, m)
18	4.33 (1H, m)	4.33 (1H, m)	47-NH	7.98 (1H, m)	7.9 8(1H, m)
18-NH	7.98 (1H, m)	7.98 (1H, m)	48	3.45 (1H, t, 8.52)	3.45 (1H, t, 8.24)
19	1.70 (1H, m)	1.70 (1H, m)		3.60 (1H, m)	3.60 (1H, m)
	1.88 (1H, m)	1.88 (1H, m)	48-NH	8.15 (1H, d, 4.76)	8.15 (1H, d, 5.16)
20	2.07 (2H, m)	2.06 (2H, m)			
			49-NH	7.74 (1H, m)	7.74 (1H, m)
21-NH_2	6.77 (1H, br s)	6.77 (1H, br s)			
	7.25 (1H, br s)	7.25 (1H, br s)	51	4.01 (1H, t, 9.76)	4.01(1H, t, 9.84)
			51-NH	8.75 (1H, d, 9.96)	8.75 (1H, d, 9.76)
23	4.38 (1H, m)	4.39 (1H, m)	52	1.88 (1H, m)	1.88 (1H, m)
23-NH	7.77 (1H, m)	7.76 (1H, d, 9.36)	53	0.78 (3H, m)	0.78 (3H, m)
24	1.80 (1H, m)	1.80 (1H, m)	54	1.15 (1H, m)	1.15 (1H, m)
25	0.77 (3H, m)	0.77 (3H, m)		1.45 (1H, m)	1.45 (1H, m)
26	1.07 (1H, m)	1.07 (1H, m)	55	0.80 (3H, m)	0.80 (3H, m)
	1.27 (1H, m)	1.27 (1H, m)			
27	0.80 (3H, m)	0.80 (3H, m)			

Supplementary Table 5 ^{13}C NMR data of teixobactin

entry	Compound **15**	teixobactin[a]	entry	Compound 15	teixobactin[a]
1	31.19	31.18	30	37.08	37.07
2	61.15	61.14	31	15.43	15.42
3	35.77	35.75	32	24.23	24.22
4	134.66	134.65	33	11.50	11.49
5,5’	129.27	129.26	34	170.55	170.55
6,6’	128.47	128.47	35	55.17	55.15
7	127.06	127.05	36	63.77	63.76
8	166.61	166.61	37	171.40	171.39
9	57.48	57.49	38	55.67	55.67
10	36.43	36.41	39	70.23	70.22
11	15.03	15.02	40	15.50	15.50
12	23.81	23.80	41	167.92	167.92
13	10.94	10.94	42	51.79	51.79
14	170.14	170.13	43	16.76	16.75
15	55.23	55.23	44	172.51	172.51
16	61.88	61.88	45	52.07	52.07
17	169.66	169.66	46	36.05	36.04
18	52.07	52.07	47	53.38	53.38
19	28.16	28.14	48	47.95	47.94
20	31.46	31.45	49	158.96	158.96
21	173.94	173.94	50	172.51	172.51
22	171.05	171.04	51	57.23	57.23
23	55.53	55.52	52	35.42	35.41
24	37.03	37.01	53	15.06	15.06
25	14.21	14.20	54	24.93	24.92
26	25.82	25.81	55	10.07	10.06
27	11.17	11.16	56	169.23	169.23
28	170.76	170.75			
29	56.80	56.79			

a) The sample of teixobactin is provided by Novobiotic Pharmaceuticals as control.

Supplementary Methods

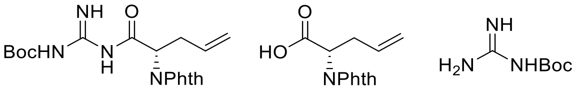

4a SM-1 SM-2

Supplementary Figure 55: Structure of compounds 4a, SM-1 and SM-2

Synthesis of 4a: SM-1(735 mg, 3 mmol) and BOP (1.59 g, 3.6 mmol) was dissolved in DMF (15 ml), NMM (1 ml, 9 mmol) was added, the mixture was stirred at room temperature for 20 min, then SM-2 (954 mg, 6 mmol) was added. The reaction was allowed to stir at room temperature overnight. After completion, the reaction mixture was diluted with EA (50 ml), washed with water (100ml x 1), 1M HCl (100 ml x 3) and brine (100 ml x 1). The organic phase was collected and dried over anhydrous Na_2SO_4, concentrated under vacuum and purified by flash column chromatography on silica gel (PE/EA, 2:1) to give compound **4a** in 80% yield as white solid (926 mg). ^{1}H NMR (400 MHz, $CDCl_3$) δ (ppm) 8.53 (s,1H), 7.82-7.80 (m, 2H), 7.70-7.68 (m, 2H), 5.72-5.62 (m, 1H), 5.00-4.96 (d, J = 17 Hz, 1H),4.92-4.90 (d, J = 10.12 Hz, 1H), 4.83-4.79 (dd, J_1 = 4.36 Hz, J_2 = 11.24 Hz, 1H), 2.90-2.76 (m, 2H), 1.48 (s,9H). ^{13}C NMR (400 MHz, $CDCl_3$) δ 181.38, 168.12, 168.02, 159.43, 159.30, 153.49, 134.57, 134.02, 131.97, 131.89, 123.33, 117.78, 83.85, 55.89, 33.23, 27.98. LRMS (ESI) calculated for $C_{19}H_{22}N_4O_5$ $[M + H]^+$ 387.16, found 387.39.

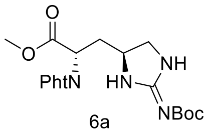

6a

Supplementary Figure 56: Structure of compound 6a

Synthesis of compound 6a compound **4a** (38.6 mg, 0.1 mmol) was dissolved in MeCN, $NaHCO_3$ (33.6 mg, 0.4 mmol) was added in ice bath, and then, I_2 (45 mg, 0.3 mmol) was added in portion. After stirring at 0 °C for 30 min. The reaction was concentrated in vacuum immediately and MeOH:AcOH 9:1 was added. Then the reaction was stirred at room temperature for 15 min. After completion, the mixture was treated with $NaHCO_3$ saturated aqueous solution. The organic phase was collected and dried over anhydrous Na_2SO_4, concentrated under vacuum and purified by flash column chromatography on silica gel (DCM:MeOH/10:1) to give compound **6a** in 65% yield. ^{1}H NMR (400 MHz, $CDCl_3$) δ (ppm) 7.88-7.86 (m, 2H), 7.77-7.75 (m, 2H), 5.02-4.98 (m, 1H), 4.35-4.30 (m, 1H), 4.20-4.15 (t, J = 10.08 Hz, 1H), 3.73 (s, 3H), 3.72-3.67 (m, 1H), 2.74-2.67 (m, 1H), 2.51-2.44 (m, 1H), 1.51 (s, 9H) . ^{13}C NMR (400 MHz, $CDCl_3$) δ 168.58, 167.62, 156.70, 134.75, 131.67, 124.15, 87.26, 53.41, 51.12, 50.21, 48.60, 34.75, 28.01. LRMS (ESI) calculated for $C_{20}H_{24}N_4O_6$ $[M + H]^+$ 417.17, found 417.41.

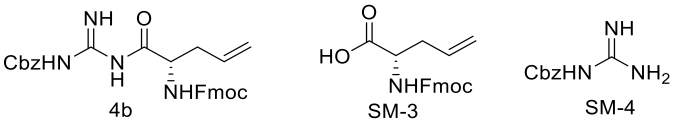

4b SM-3 SM-4

Supplementary Figure 57: Structure of compound 4b, SM-3a and SM-4

Step 1 : Synthesis of SM-3. 2-aminopent-4-enoic acid (11.5 g, 100 mmol) was dissolved in acetone: H_2O/ 1:1 (200 ml), FmocOSu (37.07 g, 110 mmol) and $NaHCO_3$ (42 g, 500 mmol) were added. The reaction was stirred at RT overnight. After completion, the mixture was evaporated under reduced pressure to remove acetone, and 1M HCl was added in drop until PH 3 then aqueous phase was extracted with EA. The organic phase was dried over anhydrous Na_2SO_4 and concentrated under vacuum to afford **SM-3** quantitatively without further purification.

Step 2 : Synthesis of SM-4. Add guanidine monohydrochloride (9.55 g, 100 mmol) to a solution of NaOH

(4.8 g, 120 mmol) in H_2O (50 mL), the aqueous solution was stirred at 0 °C for 10 minutes. The solution of CbzCl (2.82 ml, 20 mmol) in acetone (50 ml) was added to the aqueous solution above at 0 °C. The mixture was stirred at room temperature for 2 h. After the addition was completed, the mixture was evaporated under reduced pressure to remove acetone and the aqueous phase was extracted with EA (a small amount of THF could be added). The organic phase was dried over anhydrous Na_2SO_4 and concentrated under vacuum. When white solid was precipitated, the mixture was filtered with anhydrous ether, and the **SM-4** was collected as white solid without purification.

Step 3: Synthesis of compound 4b. **SM-3** (13.48 g, 40 mmol) and BOP (20.26 g, 48 mmol) were dissolved in DMF (200 ml), NMM (13.17 ml, 120 mmol) was added, the mixture was stirred at room temperature for 20 min, then compound **SM-4** (15.44 g, 80 mmol) was added. The reaction was allowed to stir at room temperature overnight. After completion, the reaction mixture was diluted with EA (600 mL), washed with water (600 mL), 1M HCl (500 mL) and brine (600 mL x 1). The organic phase was collected and dried over anhydrous Na_2SO_4, concentrated under vacuum and purified by flash column chromatography on silica gel (PE/EA, 2:1) to give compound **4b** (16.4 g) in 80% yield as white solid. ^{1}H NMR (400 MHz, $CDCl_3$) δ (ppm) 7.76-7.75 (d, J = 7.6 Hz, 2H), 7.61-7.57 (m, 2H), 7.41-7.37 (m, 2H), 7.31-7.26 (m, 7H), 5.68 (s, 1H), 5.29-5.11 (m, 4H), 4.47-4.40 (m, 2H), 4.22-4.13 (m, 2H), 2.60-2.59(m,1H), 2.50-2.47 (m, 1H). ^{13}C NMR (400 MHz, $CDCl_3$) δ 158.85, 156.05, 143.99, 143.78, 141.38, 135.72, 132.34, 128.61, 128.40, 128.28, 127.80,127.14, 125.19, 120.06, 119.42, 67.57, 67.26, 55.97, 47.22, 36.77. LRMS (ESI) calculated for $C_{29}H_{28}N_4O_5$ $[M + H]^+$ 513.21, found 513.75.

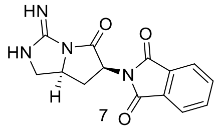

7

Supplementary Figure 58: Structure of compound 7

Synthesis of compound 7.Compound **4a** (39 mg, 0.1 mmol) was dissolved in MeCN, $NaHCO_3$ (34 mg, 0.4 mmol) was added at 0 °C. I_2 (102 mg, 0.4 mmol) was added in portion. The reaction was stirred at room tempreture for half an hour, then the solent was evaporated and TFA was added. The reaction was stirred for 30 min and TFA was evaporated. The residue was purified by HPLC to get desired compound **7**. ^{1}H NMR (400 MHz, CD_3OD) δ (ppm) 7.92-7.85 (m, 4H), 5.62-5.56 (dd, J = 11.6 Hz, J = 8.4 Hz, 1H), 4.89-4.77 (m, 1H), 4.07 (dd, J = 10.4 Hz, J = 8.8 Hz, 1H), 3.68 (t, J = 9.6 Hz, 1H), 2.92-2.86 (m, 1H), 2.85-2.62 (m, 1H).

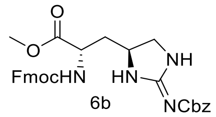

6b

Supplementary Figure 59: Structure of compound 6b

Synthesis of compound 6b. Compound **4b** (32.03 g, 62.6 mmol) was dissolved in 200 mL THF, $NaHCO_3$ (31.56 g, 376 mmol) was added at 0 °C. I_2 (56.25 g, 250.4 mmol) was added in portion. The reaction was stirred at 0 °C for one hour and detected by LC-MS. After completion, the reaction mixture was concentrated under vacuum. And then, MeOH:AcOH 9:1 (500 ml) was added, and the mixture was stirred at room temperature for 15 min, and then $NaHCO_3$ aqueous solution and $Na_2S_2O_3$ aqueous solution was added until the solution is clear and free of bubbles. The organic layer was evaporated and saturated, the production was extracted by DCM and dried over anhydrous Na_2SO_4, concentrated under vacuum and purified by flash column chromatography on silica gel (DCM:MeOH/20:1) to give compound **6b** (22.4 g) in 66% yield. ^{1}H NMR (400 MHz, DMSO) δ (ppm) 7.90-7.88 (d, J = 7.48 Hz, 2H), 7.82-7.80 (d, J = 7.8 Hz, 1H), 7.71-7.69 (m, 2H),7.43-7.31 (m, 8H), 5.21 (s,2H), 4.36-4.26 (m, 3H), 4.22-4.21 (m, 1H), 3.97-3.92 (t, J = 9.48Hz, 1H), 3.87-3.80 (m,

1H), 3.62 (s,3H), 3.51-3.47 (m, 1H), 1.86-1.82 (m, 2H). ^{13}C NMR (400 MHz, DMSO) δ 172.80, 156.04, 151.92, 143.72, 140.70, 139.38, 137.39, 135.59, 128.89, 128.45, 128.36, 128.21, 127.83, 127.61, 127.26, 127.05, 125.16, 124.87, 121.35, 120.11, 120.00, 109.73, 67.30, 65.62, 51.94, 51.50, 50.68, 46.61, 37.32. LRMS (ESI) calculated for $C_{30}H_{30}N_4O_6$ $[M + H]^+$ 543.22, found 543.86 .

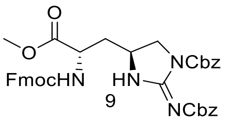

Supplementary Figure 60: Structure of compound 9

Synthesis of compound 9 Compound **6b** (19 g, 35.05 mmol) was dissolved in DCM**,** DIEA (23.1 ml, 140.2 mmol) was added. CbzOSu (26.2 g, 105.15 mmol) was added in portion. The reaction was stirred at room temperature for 5 h. After completion, the mixture was diluted with DCM and washed with 1M HCl, aqueous saturated $NaHCO_3$, and brine. Then the organic layer was dried with Na_2SO_4, filtered and concentrated. The product was purified by silica gel column with PE/EA (1:2) to give compound **9** (19 g, 28 mmol) in 80%. ^{1}H NMR (400 MHz, DMSO) δ (ppm) 9.02(s,1H), 7.90-7.88 (m,3H), 7.72-7.70 (d, J = 7.40 Hz, 2H), 7.48-7.47 (d, J = 6.52 Hz, 2H), 7.41-7.30 (m, 13 H), 5.21 (s, 2H), 5.06 (s, 2H), 4.37-4.35 (m, 2H), 4.25-4.22 (m, 2H), 3.98-3.95 (t, J = 9.68 Hz, 1H), 3.84-3.81 (m, 1H), 3.63 (s, 4H), 2.11-2.07 (m, 1H), 1.87-1.86(m, 1H). ^{13}C NMR (400 MHz, DMSO) δ 172.03, 162.78, 157.87, 156.07, 150.30, 143.71, 143.69, 140.74, 137.22, 135.89, 128.26, 127.82, 127.76, 127.66, 127.59, 127.43,127.05, 126.99,125.19, 125.13,120.09, 67.07, 66.17, 65.62, 52.06, 51.10, 49.05, 46.68, 36.31. LRMS (ESI) calculated for $C_{38}H_{36}N_4O_8$ $[M + H]^+$ 677.25, found 677.91.

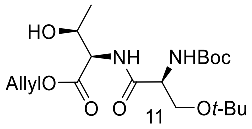

Supplementary Figure 61: Structure of compound 11

Synthesis of compound 11 Compound **10** (16.85 g, 65 mmol) was dissolved in 100 ml 30 % TFA in DCM and stirred for 30 min at RT. After completion, the solvent was evaporated to get the residue without purification. Boc-Ser(tBu)-OH (20.4 g, 78 mmol), HATU (26.6 g, 78 mmol) and DIEA (32.2 mL, 195 mmol) were dissolved in DCM/DMF 2:1 (100 mL), then the residue was added and stirred at room temperature for 3 h. The solvent was evaporated and the mixture was diluted with EA (100 mL) and washed with 1M HCl (100 mL), aqueous saturated $NaHCO_3$ (100 mL), water (100ml) and brine (100 mL). Then the organic layer was dried with Na_2SO_4 and concentrated to give compound **11** without purification. ^{1}H NMR (400 MHz, $CDCl_3$) δ (ppm) 7.26-7.23 (d, J = 11.6 Hz, 1H), 5.87-5.81 (m, 1H), 5.47 (s, 1H), 5.30-5.29 (d, J = 1.2 Hz, 1H), 5.25-5.24 (d, J_1 = 1.6 Hz, 1H), 4.60-4.58 (d, J =5.6 Hz, 2H), 4.56-4.53 (m,1H), 4.27 (s, 1H), 4.18 (s, 1H), 3.73-3.72 (m, 1H), 3.42-3.38 (m, 1H), 1.39 (s, 9H), 1.19-1.13 (m, 12H). ^{13}C NMR (400 MHz, $CDCl_3$) δ 171.22, 170.48, 155.65, 131.58, 119.03, 118.94, 80.19, 74.17, 68.25, 66.16, 61.77, 57.51, 54.91, 28.41, 27.46, 20.02. LRMS (ESI) calculated for $C_{19}H_{34}N_2O_7$ $[M + H]^+$ 403.49, found 403.83.

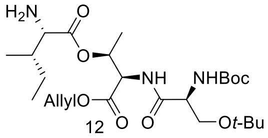

Supplementary Figure 62: Structure of compound 12

Synthesis of compound 12 Fmoc-Ile-OH (34.5 g, 97.5 mmol), EDCI (18.7 g, 97.5 mmol), DMAP (1.59 g, 13 mmol) was dissolved in 200 mL DCM and the mixture was stirred for 20 min. Then compound **11** (26 g, 65

mmol) was added to the solution and stirred at room temperature for 12 h. The mixture was diluted with DCM (100 mL) and washed with aqueous saturated $NaHCO_3$ (100 mL) and brine (100 mL). Then the organic layer was dried with Na_2SO_4 and concentrated. Then the intermediate was dissolved in 100 mL 33% diethylamine in MeCN and stirred for 15 min at RT. After completion, the solvent was evaporated and purified by flash column chromatography on silica gel (DCM/MeOH, 20:1) to afford compound **12** (26.5 g, 51 mmol) in 79%.^{1}H NMR (400 MHz, $CDCl_3$) δ (ppm) 7.26-7.20 (s, 1H), 5.92-5.82 (m, 1H), 5.50-5.44 (m, 2H), 5.33-5.29 (d, *J* = 17.24 Hz, 1H), 5.26-5.23 (d, *J* = 9.36 Hz, 1H), 4.86-4.83 (d, *J* = 10.36 Hz, 1H), 4.64-4.52 (m, 2H), 4.24 (s, 1H), 3.80 (s, 1H), 3.46-3.42 (m, 1H), 3.24-3.23 (d, *J* = 4.84 Hz, 1H), 1.68-1.67 (s, 1H), 1.60 (s, 1H), 1.45 (s, 9H), 1.29-1.28 (d, *J* = 6.40 Hz, 3H), 1.20 (s, 9H), 1.19-1.17 (m, 2H), 0.90-0.87 (m, 6H). ^{13}C NMR (400 MHz, $CDCl_3$) δ 174.46, 171.36, 169.05, 155.65, 131.35, 119.28, 80.19, 74.20, 71.28, 66.40, 61.72, 59.68, 55.52, 54.82, 38.72, 28.44, 27.52, 24.18, 17.05, 15.79, 11.68. LRMS (ESI) calculated for $C_{25}H_{45}N_3O_8$ $[M + H]^+$ 516.33, found 516.97.

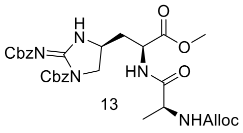

Supplementary Figure 63: Structure of compound 13

Synthesis of compound 13 Compound **9** (4.9 g, 7.25 mmol) was dissolved in 60 ml 33% diethylamine in MeCN and stirred for 15 min at RT. After completion, the solvent was removed for next step without further purification. Alloc-Ala-OH (2.6 g, 15 mmol), HATU (5.7 g, 15 mmol) and DIEA (2.5 mL, 15 mmol) were mixed in 80 mL DMF:DCM (1:4). Then the intermediate was added to the above solution. The reaction mixture was stirred at room temperature for 3 h. the solvent was removed and dissolved in ethyl acetate. Then the solution was washed with aqueous saturated $NaHCO_3$ (20 ml x 2), 1M HCl (20 ml) and brine, dried over anhydrous Na_2SO_4, concentrated under vacuum and purified by flash column chromatography on silica gel (DCM/MeOH, 20:1) to afford compound **13** (3.13 g) in 71% isolated yield. ^{1}H NMR (400 MHz, $CDCl_3$) δ (ppm) 7.42-7.34 (m, 10H), 5.91-5.87 (m, 1H), 5.73-5.71 (d, *J* = 7.2 Hz, 1H), 5.31-5.27 (m, 1H), 5.24-5.17 (m, 5H), 4.83-4.82 (m, 1H), 4.57-4.55 (d, *J* = 5.32 Hz, 2H), 4.39-4.36 (m, 1H), 4.13-4.10 (m, 1H), 3.74 (s, 3H), 3.47-3.45 (m, 1H), 2.22-2.20 (m, 1H),1.87-1.89 (m, 1H), 1.43-1.41 (d, *J* = 6.8 Hz, 3H). ^{13}C NMR (400 MHz, $CDCl_3$) δ 173.23, 171.72, 155.86, 152.27, 135.75, 134.88, 133.00, 128.87, 128.62, 128.43, 117.56, 68.76, 68.13, 65.70, 60.52, 52.83, 50.65, 50.46, 50.32, 37.95, 19.07. LRMS (ESI) calculated for $C_{30}H_{35}N_5O_9$ $[M + H]^+$ 610.25, found 610.84.

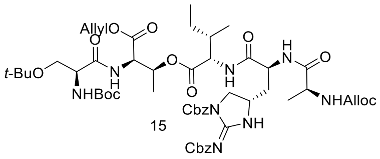

Supplementary Figure 64: Structure of compound 15

Synthesis of compound 15 Compound **13** (3.7 g, 6.08 mmol) was dissolved in 20 mL THF/H_2O (3:1). LiOH (219 mg, 9.1 mmol) was added to the above solution and stirred at ice bath for 2 min. Then additional LiOH (73 mg, 3.04 mmol) was further added to the above solution and stirred at ice bath for 2 min. After completion, the mixture was acidified to pH 3 with 1M HCl and extracted with mixture of EA and THF (EA:THF/1:1, 100 mL). The combined organic phase was dried over Na_2SO_4 and concentrated under vacuum to give intermediate without further purification. The intermediate (6 g, 10 mmol) and compound **12** (12.9 g, 25 mmol) were mixed in100 mL THF/DMF (1:1). DIEA (4.1 mL, 25 mmol) and DEPBT (6 g, 20 mmol) were added at 0 ℃. Then the reaction was stirred at room temperature overnight. The reaction mixture was washed with 1 M HCl and

brine. The organic phase was dried over Na_2SO_4, concentrated under vacuum and purified by flash column chromatography on silica gel (DCM/MeOH, 20:1) to give compound **15** (6.9 g) in 63%.^{1}H NMR (400 MHz, $CDCl_3$) δ (ppm) 7.43-7.38 (m, 4H), 7.33-7.28 (m, 6H), 5.92-5.86 (m, 2H), 5.48 (s,1H), 5.31 (s,1H), 5.27-5.26 (m, 1H), 5.23-5.22 (m, 2H), 5.20-5.15 (m, 3H), 4.69-4.68 (m, 1H), 4.57 (s, 2H), 4.52 (s, 2H), 4.32 (s, 1H), 4.31-4.30 (m, 1H), 4.27-4.23 (m, 1H), 4.20-4.18 (m, 1H), 4.04-4.03 (m, 2H), 3.68-3.66 (m, 1H), 3.61-3.59 (m, 2H), 2.10 (s, 1H), 1.91-1.89 (m, 2H), 1.44 (s, 9H), 1.38-1.35 (m, 5H), 1.26-1.25 (m, 3H), 1.17 (s, 9H), 0.91-0.90 (m, 6H). ^{13}C NMR (400 MHz, $CDCl_3$) δ 175.92, 173.75, 173.08, 171.27, 169.97, 158.18, 157.38, 138.01, 136.85, 134.22, 133.01, 129.56, 129.39, 129.29, 129.12, 128.97, 119.00, 118.70, 117.59, 80.74, 74.76, 72.79, 69.22, 68.34, 67.25, 67.18, 66.92, 66.90, 66.86, 66.58, 63.23, 58.81, 58.58, 56.70, 56.62, 56.36, 54.80, 52.31, 51.53, 50.81, 38.57, 37.83, 28.71, 27.73, 25.93, 18.07, 17.21, 16.30, 16.27, 16.23, 16.02, 15.89, 11.87. LRMS (ESI) calculated for $C_{54}H_{76}N_8O_{16}$ $[M + H]^+$ 1093.54, found 1094.41.

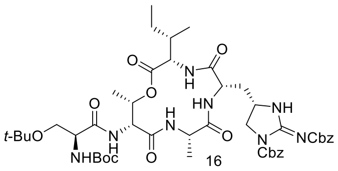

16

Supplementary Figure 65: Structure of compound 16

Synthesis of compound 16 Compound **15** (8.85 g, 8.1 mmol), $Pd(PPh_3)_4$ (2.78 g, 2.4 mmol), 1,3-dimethylbarbituric acid (2.5 g, 16.2 mmol) was added under the protection of Ar. Anhydrous DCM (50 ml) was added, the reaction was stirred at RT for 1h. After completion, the mixture above was diluted by DCM:DMF(4:1) 4 L. Then HOAT (4.46 g, 32.4 mmol), HATU (12.5 g, 32.4 mmol) and DIEA (10.8 mL, 65.6mmol) were dissolved in DMF (50 ml), and added to the mixture above. The reaction was stirred at ice bath for 10 min, and then moved to RT for 24 h. After completion, the solvent was removed and dissolved in ethyl acetate. Then the solution was washed with aqueous saturated $NaHCO_3$, 1M HCl and brine, dried over anhydrous Na_2SO_4, concentrated under vacuum and purified by flash column chromatography on silica gel (DCM/MeOH, 10:1) to afford compound **16** (4.46 g, 4.7 mmol) in 58% yield. ^{1}H NMR (400 MHz, $CDCl_3$) δ (ppm) 7.42-7.28 (m, 10H), 5.58-5.56 (m, 1H), 5.27-5.22 (m, 2H), 5.19-5.14 (m, 2H), 4.62-4.59 (m, 1H),4.23-4.21 (d, J = 8.12 Hz, 1H), 4.13-4.11 (m, 2H), 4.09-4.04 (m, 2H), 3.66-3.60 (m, 3H), 3.31-3.30 (m, 2H), 2.16 (m, 1H), 2.07 (m, 1H), 1.73 (s, 1H), 1.44 (s, 9H), 1.41-1.40 (m, 3H), 1.32-1.30 (d, J = 6.52 Hz, 3H), 1.19 (s, 9H), 0.91-0.85 (m, 6H). ^{13}C NMR (400 MHz, $CDCl_3$) δ 175.56, 175.30, 172.89, 169.50, 137.91, 136.85, 129.62, 129.46, 129.08, 81.35, 74.89, 72.66, 70.52, 69.40, 68.48, 62.03, 61.52, 59.53, 53.73, 53.56, 50.56, 28.73, 27.69, 26.58, 20.58, 16.90, 16.82, 15.57, 14.46, 11.34. LRMS (ESI) calculated for $C_{47}H_{66}N_8O_{13}$ $[M + H]^+$ 951.47, found 952.00.

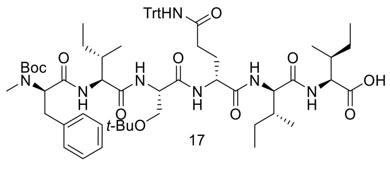

17

Supplementary Figure 66: Structure of compound 17

Synthesis of compound 17 4 g of 2-Cl-Trt resin (1 mmol/g) was placed in a 100 mL polypropylene syringe. After activation, the first amino acid Fmoc-L-Ile-OH (3.5 g, 10 mmol) and DIEA (6.6 mL, 40 mmol) in 40mL DCM/DMF (1:1) and shacked for 4 h. Then the unreacted Cl group was blocked with isopropanol/DIEA/DMF (1:1:8) for 10 minutes. Next, the resin was washed with DMF and DCM. Then Fmoc was removed by 20 % piperidine in DMF. The next amino acids were added by using the following coupling condition: For coupling conditions: Fmoc-AA-OH/HATU/DIEA (3:3:6) in DMF for 50 min. The following amino acids were coupled

one by one: Fmoc-D-*allo*-Ile-OH, Fmoc-D-Gln(Trt)-OH, Boc-Ser(*t*Bu)-OH, Fmoc-Ile-OH, Boc-NMe-D-Phe-OH. At last, desired linear hexapeptide was cleaved from the resin with 20% TFE (in DCM). The filtrate was concentrated under reduced pressure to afford compound **17** (4.3 g, 95%).

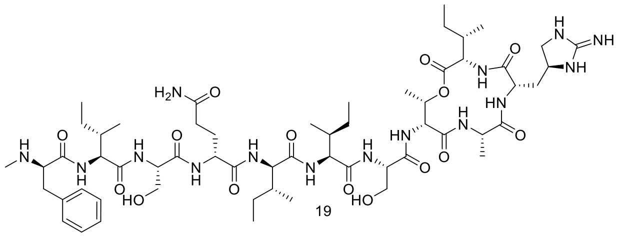

Supplementary Figure 67: Structure of compound 19

Synthesis of compound 19 (teixobactin). Compound **16** (2.85 g, 3 mmol) was dissolved in 30 ml 3 M HCl in dioxane and stirred for 15 min. when the reaction finished, the protecting group Boc was removed completely, then the solvent was evaporated and the residue was diluted with cosolvent (EA/THF) and washed with aqueous saturated NaCl, $NaHCO_3$. The organic phase was dried over Na_2SO_4 and concentrated under vacuum without purification. Then the intermediate and compound **17** (3.4 g, 3 mmol) were dissolved in DMF. DEPBT (1.08 g, 3.6 mmol) and DIEA (594 µL, 3.6 mmol) were added at ice bath and stirred at room tempreture overnight. The mixture was diluted with EtOAc and washed with 1M HCl, aqueous saturated $NaHCO_3$, and brine. Then the organic layer was dried with Na_2SO_4 and concentrated to give compound **18** without purification. To a solution of compound **14** in MeOH/HCOOH (9:1) was added $Pd(OH)_2/C$ (20% w/w, 850 mg), and the reaction mixture was stirred at room tempreture under H_2 (1 atm) for 1h. Then the reaction mixture was filtrated and concentrated in vacuum. Then the intermediate was treated with 20 ml TFA/H_2O/TIPS (*v/v/v* = 95:2.5:2.5) for 1 hour and the reaction was monitored by LC-MS. After completion, the reaction mixture was concentrated under low temperature, following by the addition of cold diethyl ether to precipitate the crude product. The crude product was purified by preparative HPLC (5-60% CH_3CN/H_2O over 30 min) to afford teixobactin (compound **19**) (1.16 g, 31%)

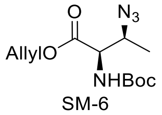

Supplementary Figure 68: Structure of compound SM-6

Synthesis of compound SM-6. To a solution of Boc-D-*allo*-Thr-OH **SM-5** (1.295 g, 5 mmol) in dry DCM (15 mL) at -78℃. Trifluoromethanesulfonic anhydride (1013 µl, 6 mmol) were sequentially added dropwise and then 2,6-lutidine (756 µl, 6.5 mmol) was added slowly. After stirring at the same temperature for 1.5 h and monitoring by TLC (Hex:EtOAc/8:2), tetrabutylammonium azide (n-Bu_4N_3) (3.56 g, 12.5 mmol) was added in portions. After stirring for 1 h at -78 ℃, the cooling bath was removed and the reaction mixture was allowed to reach 23℃ for 1.5 h. A saturated aqueous solution of $NaHCO_3$ was added, and the aqueous phase extracted with EtOAc. The crude product was purified by flash chromatography over silica gel (Hex:EtOAc 95:5 a 9:1) to get oil compound **SM-6** (1.2 g, 85% yield). ^{1}H NMR (400 MHz, $CDCl_3$) δ (ppm) 5.95-5.80 (m, 1H), 5.35-5.28 (dd, *J* = 17.2 Hz, *J* = 1.16 Hz, 1H), 5.26-5.20 (d, *J* = 10.4 Hz, 1H), 5.18-5.12 (d, *J* = 9.32 Hz, 1H), 4.68-4.58 (m, 2H), 4.38-4.30 (dd, *J* = 9.44 Hz, *J* = 2.48 Hz, 1H), 4.18-4.10 (m, 1H), 1.41 (s, 9H), 1.31 (d, *J* = 5.72 Hz, 3H).

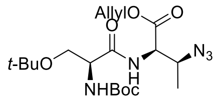

SM-7

Supplementary Figure 69: Structure of compound SM-7

Synthesis of compound SM-7. Compound **SM-6** (800 mg, 2.8 mmol) was dissolved in 15 ml 30% TFA in DCM and stirred for 15 min at RT. After removal of protecting group Boc, the solvent was evaporated to get intermediate without purification. Boc-Ser(*t*Bu)-OH (913 mg, 3.5 mmol), HATU (1.33 g, 3.5 mmol) and DIEA (1.39 ml, 8.4 mmol) were dissolved in DCM/DMF (10 ml), then the intermediate was added and stirred at room temperature for 3 h. Then the solvent was evaporated and the mixture was diluted with EA (50 ml) and washed sequentially with water (100ml), 1M HCl (30 ml), aqueous saturated $NaHCO_3$ (30 ml), and brine (30 ml). Then the organic layer was dried with Na_2SO_4 and evaporated. The product was purified by silica gel column with PE/EA (2:1) to give compound **SM-7** (956 mg, 80% for 2 steps). ^{1}H NMR (400 MHz, $CDCl_3$) δ (ppm) 7.14 (d, J = 8.92 Hz, 1H), 5.96-5.85 (m, 1H), 5.43 (s, 1H), 5.38-5.32 (dd, J = 17.16 Hz, J = 1.32 Hz,) 5.30-5.26 (dd, J = 9.52 Hz, J = 1.04 Hz, 1H), 4.80-4.73 (dd, J = 9.18 Hz, J = 2.64 Hz, 1H), 4.67 (d, J = 5.80 Hz, 1H), 4.30-4.18 (m, 2H), 3.83-3.78 (m, 1H), 3.47-3.42 (dd, J = 8.80 Hz, J = 6.72 Hz, 1H), 1.46 (s, 9H), 1.33 (d, J = 6.68 Hz, 3H), 1.20 (s, 9H).

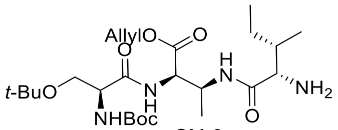

SM-8

Supplementary Figure 70: Structure of compound SM-8

Synthesis of compound SM-8. Compound **SM-7** (956 mg, 2.24 mmol) was dissolved in THF (5 ml) and H_2O (1 ml) and kept at 0 °C, followed by addition of PMe_3 (1.0 M in THF, 9 ml, 9 mmol). After that, the reaction was warmed to room temperature and stirred for another 12 h. The solvent was removed in vacuo to afford the crude intermediate with primary amine. Fmoc-Ile-OH (1059 mg, 3 mmol), HATU (1140 mg, 3 mmol) and DIEA (577 μl, 3.5 mmol) was dissolved in DCM/DMF (10ml, 8:2), then the crude intermediate with primary amine was added to the solvent and stirred for 3 h at 30 ℃. Then the solvent was evaporated and the mixture was diluted with EA (20 ml) and washed sequentially with 1M HCl, aqueous saturated $NaHCO_3$, and brine. Then the organic layer was dried with Na_2SO_4 and evaporated. The product was purified by silica gel column with DCM/MeOH (20:1) to give compound **SM-8** (412 mg, 36% for 2 steps). $C_{25}H_{46}N_4O_7$ $[M + H]^+$ 515.34, found 515.84.

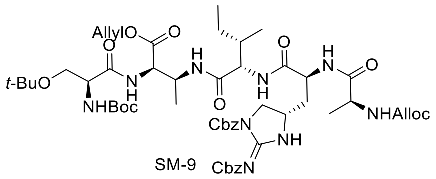

SM-9

Supplementary Figure 71: Structure of compound SM-9

Synthesis of compound SM-9. Compound **14** (480 mg, 0.8 mmol) and compound **SM-8** (308 mg, 0.6 mmol) were mixed in 10 mL THF/DMF (1:1). DEPBT (300 mg, 1 mmol) and DIEA (165 μl, 1 mmol) were added at 0 ℃. Then the reaction was stirred at room temperature overnight. The reaction mixture was washed with 1 M HCl (10 mL x 1) and brine (10 mL x 1). The organic phase was dried over Na_2SO_4, concentrated under

vacuum and purified by flash column chromatography on silica gel (DCM/MeOH, 20:1) to give compound **SM-9** (347 mg) in 53% isolated yield. ^{1}H NMR (400 MHz, $CDCl_3$) δ (ppm) 9.90 (s, 1H), 9.77 (s, 1H), 8.23 (d, *J* = 8.8 Hz, 1H), 7.46-7.30 (m, 10H), 7.08 (s, 1H), 6.78 (d, *J* = 8.8 Hz, 1H), 6.50 (s, 1H), 6.00-5.70 (m, 3H), 5.35-5.18 (m, 7H), 5.11 (d, *J* = 10.4 Hz, 1H), 4.90-4.87 (m, 1H), 4.75-4.52 (m, 4H), 4.47 (d, *J* = 5.2 Hz, 1H), 4.40-4.20 (m, 4H), 4.02-3.92 (m, 2H), 3.72-3.67 (m, 1H), 3.58-3.52 (m, 1H), 3.40-3.30 (m, 1H), 2.48 (d, *J* = 14 Hz, 1H), 2.10-2.00 (m, 1H), 1.82-1.68 (m, 1H), 1.49 (d, *J* = 7.2 Hz, 3H), 1.43 (s, 9H), 1.42-1.20 (m, 2H), 1.20-1.16 (m, 3H), 1.16 (s, 9H), 0.92-0.82 (m, 6H). $C_{54}H_{77}N_9O_{15}$ $[M + H]^+$ 1092.55, found 1093.08.

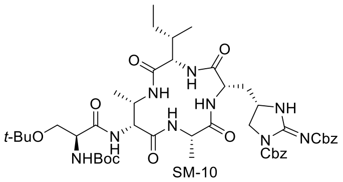

SM-10

Supplementary Figure 72: Structure of compound SM-10

Synthesis of compound SM-10. According to the synthesis of compound **16**. ^{1}H NMR (400 MHz, CD_3OD) δ (ppm) 7.46-7.23 (m, 10H), 5.30-5.18 (m, 2H), 5.13 (s, 2H), 4.68-4.52 (m, 2H), 4.20-4.11 (m, 1H), 4.08-3.92 (m, 3H), 3.77-3.53 (m, 4H), 2.23-2.12 (m, 2H), 1.92-1.82 (m, 1H), 1.60-1.50 (m, 1H), 1.50-1.40 (m, 12H), 1.22-1.12 (m, 14H), 0.92-0.82 (m, 6H). $C_{47}H_{67}N_9O_{12}$ $[M + H]^+$ 950.49, found 951.05.

Supplementary References

1. Zong, Y.; Sun, X.; Gao, H.; Meyer, K. J.; Lewis, K.; Rao, Y. Developing Equipotent Teixobactin Analogues Against Drug-Resistant Bacteria and Discovering A Hydrophobic Interaction Between Lipid II and Teixobactin. *J. Med. Chem.* **61**, 3409-3421 (2018)
2. Parmar, A. et al. Design and Syntheses of Highly Potent Teixobactin Analogues against Staphylococcus aureus, Methicillin-Resistant Staphylococcus aureus (MRSA), and Vancomycin-Resistant Enterococci (VRE) in Vitro and in Vivo. *J. Med. Chem.* **61**, 2009-2017 (2018).
