# Supplementary material for: Gram-scale total synthesis of teixobactin promoting binding mode study and discovery of more potent antibiotics
Source: Nat Commun. 2019 Jul 22;10:3268. doi: 10.1038/s41467-019-11211-y (PMC6646333; doi:10.1038/s41467-019-11211-y)
Supplement: Supplementary file 3 — Reporting Summary [file 41467_2019_11211_MOESM3_ESM.pdf]

nature research

Corresponding author(s): Yu Rao

Last updated by author(s): Feb 23, 2019

Reporting Summary

Nature Research wishes to improve the reproducibility of the work that we publish. This form provides structure for consistency and transparency in reporting. For further information on Nature Research policies, see Authors & Referees and the Editorial Policy Checklist.

Statistics

For all statistical analyses, confirm that the following items are present in the figure legend, table legend, main text, or Methods section.

n/a	Confirmed	
☐	☒	The exact sample size (*n*) for each experimental group/condition, given as a discrete number and unit of measurement
☐	☒	A statement on whether measurements were taken from distinct samples or whether the same sample was measured repeatedly
☐	☒	The statistical test(s) used AND whether they are one- or two-sided<br>*Only common tests should be described solely by name; describe more complex techniques in the Methods section.*
☒	☐	A description of all covariates tested
☒	☐	A description of any assumptions or corrections, such as tests of normality and adjustment for multiple comparisons
☒	☐	A full description of the statistical parameters including central tendency (e.g. means) or other basic estimates (e.g. regression coefficient) AND variation (e.g. standard deviation) or associated estimates of uncertainty (e.g. confidence intervals)
☐	☒	For null hypothesis testing, the test statistic (e.g. *F*, *t*, *r*) with confidence intervals, effect sizes, degrees of freedom and *P* value noted<br>*Give P values as exact values whenever suitable.*
☒	☐	For Bayesian analysis, information on the choice of priors and Markov chain Monte Carlo settings
☒	☐	For hierarchical and complex designs, identification of the appropriate level for tests and full reporting of outcomes
☒	☐	Estimates of effect sizes (e.g. Cohen's *d*, Pearson's *r*), indicating how they were calculated

Our web collection on statistics for biologists contains articles on many of the points above.

Software and code

Policy information about availability of computer code

Data collection	1H and 13C NMR spectra were recorded on a Bruker AVANCE III 400 MHz spectrometer. Low-resolution mass spectral analyses were performed with Waters AQUITY UPLCTM/MS. High-resolution mass spectral analyses were performed with Q Exactive. Cell viability data were collected with SpectraMax Plus Microplate Reader (SoftMax® Pro 6). CD spectrum and data was collected with Circular Dichroism Spectrometer, model Chirascan Plus.
Data analysis	Compound NMR data were analyzed with TopSpin 3.5pl7 and the structures of these compounds were drawn with ChemBioDraw Ultra 14.0. Cell viability data were performed with Graphpad Prism 7.0. In in vivo assay, the probability was determined by non-parametric log-rank test. It was analyzed by GraphPad Prism 5.01. Molecular docking was conducted with Schrödinger Suites Release 2017-1 and Pymol 1.7.

For manuscripts utilizing custom algorithms or software that are central to the research but not yet described in published literature, software must be made available to editors/reviewers. We strongly encourage code deposition in a community repository (e.g. GitHub). See the Nature Research guidelines for submitting code & software for further information.

Data

Policy information about availability of data

All manuscripts must include a data availability statement. This statement should provide the following information, where applicable:

- Accession codes, unique identifiers, or web links for publicly available datasets
- A list of figures that have associated raw data
- A description of any restrictions on data availability

No restrictions apply on data availability. All figures and tables have associated raw data, these are: Fig. 1-8 and Table 1-2. All other data supporting the findings of this study are available from supplementary information or from the corresponding author upon request.

Field-specific reporting

Please select the one below that is the best fit for your research. If you are not sure, read the appropriate sections before making your selection.

☒ Life sciences ☐ Behavioural & social sciences ☐ Ecological, evolutionary & environmental sciences

For a reference copy of the document with all sections, see nature.com/documents/nr-reporting-summary-flat.pdf

Life sciences study design

All studies must disclose on these points even when the disclosure is negative.

Sample size	The sample size (n) of each experiment was provided in the figure/table legends in the main manuscript and in supporting information.
Data exclusions	No data were excluded from the analyses.
Replication	All the experimental findings were reliably reproduced.
Randomization	Samples were randomly assigned by independent persons.
Blinding	In cell viability assay, the performers were blinded to the samples.

Reporting for specific materials, systems and methods

We require information from authors about some types of materials, experimental systems and methods used in many studies. Here, indicate whether each material, system or method listed is relevant to your study. If you are not sure if a list item applies to your research, read the appropriate section before selecting a response.

Materials & experimental systems

n/a	Involved in the study	
☒	☐	Antibodies
☐	☒	Eukaryotic cell lines
☒	☐	Palaeontology
☐	☒	Animals and other organisms
☒	☐	Human research participants
☒	☐	Clinical data

Methods

n/a	Involved in the study	
☒	☐	ChIP-seq
☒	☐	Flow cytometry
☒	☐	MRI-based neuroimaging

Eukaryotic cell lines

Policy information about cell lines

Cell line source(s)	The HepG2 cell line was kindly provided by Prof. Mengying Lu (302 hospital).
Authentication	Authentication was performed by STR profiling.
Mycoplasma contamination	cell linewas confirmed as mycoplasma-free, both in provider's test and in the lab to carry out the study.
Commonly misidentified lines (See ICLAC register)	No misidentified cell lines were used in the study.

Animals and other organisms

Policy information about studies involving animals; ARRIVE guidelines recommended for reporting animal research

Laboratory animals	C57BL/6J female mice and Balb/c female mice were from Beijing Vital River Laboratory Animal Technology Co., Ltd. All animal experiments were done under the guidelines of Laboratory Animal Research Center, Tsinghua University and used an approved animal protocol (16-RY2, PI, Yu Rao). Northeastern IACUC approved our animal study infected with MRSA 33591.
Wild animals	No wild animals were used in this study.
Field-collected samples	No field-collected samples were used in this study.
Ethics oversight	all animal experiments were done under the guidelines of Laboratory Animal Research Center, Tsinghua University and used an approved animal protocol (16-RY2, PI, Yu Rao). Northeastern IACUC approved our animal study infected with MRSA 33591.

Note that full information on the approval of the study protocol must also be provided in the manuscript.
